# Improved inference of chromosome conformation from images of labeled loci

**DOI:** 10.12688/f1000research.16252.3

**Published:** 2019-03-28

**Authors:** Brian C. Ross, James C. Costello

**Affiliations:** 1 Computational Bioscience Program, Department of Pharmacology, University of Colorado, Anschutz Medical Campus, Aurora, CO, 80045, USA

**Keywords:** chromosome, conformation, reconstruction, fluorescence, genetic, loci

## Abstract

We previously published a method that infers chromosome conformation from images of fluorescently-tagged genomic loci, for the case when there are many loci labeled with each distinguishable color.  Here we build on our previous work and improve the reconstruction algorithm to address previous limitations.  We show that these improvements 1) increase the reconstruction accuracy and 2) allow the method to be used on large-scale problems involving several hundred labeled loci.  Simulations indicate that full-chromosome reconstructions at 1/2 Mb resolution are possible using existing labeling and imaging technologies.  The updated reconstruction code and the script files used for this paper are available at: https://github.com/heltilda/align3d.

## Introduction

Measurement of *in vivo* chromosome conformation is a major unsolved problem in structural biology despite its known biological importance ^1^. The present state-of-art is to either obtain indirect information about conformations using 3C-derived methods which measure DNA-DNA contacts (typically in a cell-averaged population) ^2^, or else directly measure the cellular locations of individual chromosomal loci in single cells by microscopy ^3^. The major limitation of direct localization is one of throughput: only ~ 3–5 labeled loci can be uniquely identified ‘by color’ in a standard microscope image, whereas a whole-chromosome reconstruction would involve labeling and identifying hundreds or thousands of loci.

Several research efforts aim to remove the color limitation either by experimental improvements or computational inferences. The experimental approaches aim to allow an increased number of labels that can be distinguished in an image ^4– 6^. Alternatively, attempts have been made to infer the identity of labels that cannot be uniquely identified in an image, by comparing the image to the known label positions along the DNA contour. The first attempt to do this was ‘by eye’ ^7^, but subsequently two computational algorithms have been developed to automate this inference: align3d ^8^ and ChromoTrace ^9^. There are two important differences between these algorithms. First, align3d has less stringent experimental requirements than ChromoTrace, as it allows for missing labels in the image and does not require a uniform label spacing along the chromosome. Second, ChromoTrace outputs explicit conformations, whereas align3d outputs likelihoods of the various possible identities for each labeled locus. Both approaches have their advantages: ChromoTrace output is straightforward to interpret, whereas align3d output gives information on the range of possible conformational solutions along with their likelihoods.

**Figure 1.**
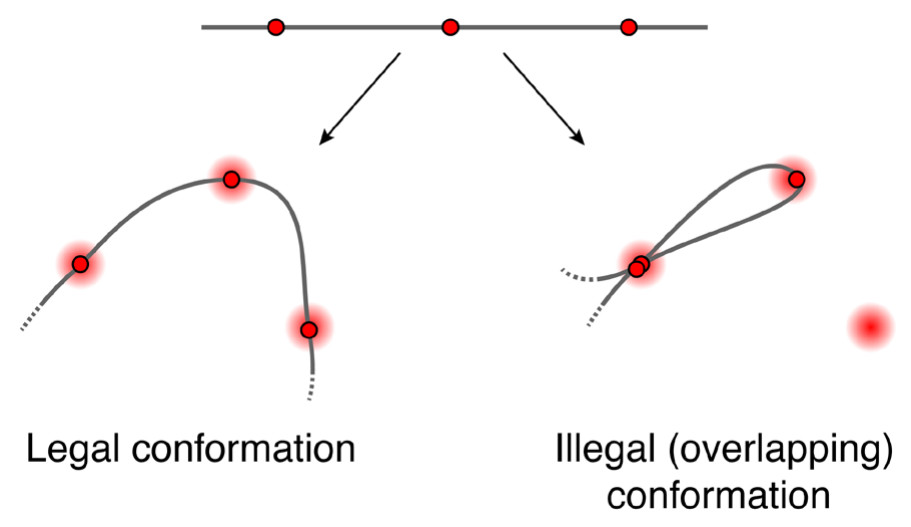
Legal versus illegal (overlapping) conformations. Schematic showing one legal and one illegal conformation passing through spots *A*, *B* and *C*. align3d counts both legal and overlapping conformations in estimating the partition *Z* (although it is able to prevent *adjacent* loci from overlapping).

This paper presents improvements to align3d ^8^ that allow it to generate high-quality, chromosome-scale conformational reconstructions. First, we briefly describe the algorithm. Using a) the genomic locations and colors of labeled loci and b) the spatial locations and colors of spots in a microscope image, together with a relation tying the genomic distance between two loci to their average spatial displacement, this method constructs a table of ‘mapping probabilities’ *p*( *L* → *s*) for a given labeled genomic locus *L* having produced spot *s* in the microscope image. Each mapping probability *p*( *L* → *s*) is calculated by dividing the summed statistical weights of conformations where locus *L* maps to spot *s*, which we term a mapping partition function and denote *Z*_*L*→ *s*_, by the full partition function *Z* that is the summed weight of all conformations. A proper calculation of *Z*_*L*→ *s*_ and *Z* would consider all conformations having no more than one locus at any given spot in the image ^1^, similar to a traveling salesman tour ^10^, but this exact calculation is intractable for large problems. Instead, align3d counts all conformations for which *adjacent* loci do not overlap at the same spot (see Figure 1), using a variant of the forward-backward algorithm ^11^ that can propagate between non-adjacent layers. This is a major source of error as the vast majority of conformations contributing to the partition function overlap at non-adjacent loci, and one consequence is that the normalization of mapping probabilities makes no sense for a non-overlapping conformation, as ∑ _*L*_ *p*( *L* → *s*) can exceed 100% for certain spots. To recover from this error, align3d assigns a penalty to each spot and iteratively adjusts these penalties until the spot normalization is sensible. Although somewhat ad hoc, use of spot penalties recovers significant information about medium-sized conformations (∼ 30 labeled loci), although larger simulated experiments (∼ 300 loci) have convergence problems due to the cost function plateauing at very small or large values of the spot penalties.

The final step is to use the mapping probabilities to construct the range of likely conformations compatible with the microscope image. Uncertainty in the conformation results from inaccuracy or uncertainty in the mapping probabilities due to three factors: inaccuracy in the DNA model (the relation between genomic and spatial distance), error in estimating the partition functions, and the inherent uncertainty in the data even with a perfect reconstruction algorithm. The DNA model can be calibrated by a control experiment, and we argue that the remaining model error can reduce our method’s confidence in its results but it generally does *not* cause our method to reconstruct mistaken conformations. The main focus of this paper is on improving the partition function estimate, using two different strategies. First, we give an efficient method for optimizing the spot penalties when there are hundreds of spots in the image. Next, we provide formulas for the partition functions which allow them to be estimated to arbitrarily high accuracy (given enough computation time), without using spot penalties or any optimization. As we show using simulations, these two methods used individually or in tandem permit confident, chromosome-scale conformational reconstructions using existing experimental technologies.

## Methods

First we provide a method for efficiently optimizing the spot penalties regardless of the number of labeled loci. This rule guarantees that a) the rate of missing spots is as expected, and b) the mapping probabilities are properly normalized. Let *q _s_* denote the penalty attached to spot *s*; then the update rule for that spot penalty is:

$$q_{s}^{\prime }=\frac{\frac{1}{P\left(s\right)/N}-1}{\frac{1}{1-p_{fn}\left(c\right)}-1}\;\cdot \;\frac{\frac{1}{P\left(s\right)/N}-1}{\frac{1}{\min\left(1,P\left(s\right)\right)/N}-1}\cdot q_{s}\;(1)$$

where *N* is the number of loci, *P*( *s*) = ∑ _*L*_ *p*( *L* → *s*) is the total probability of mapping any locus to spot *s*, and *p _fn_*( *c*) is the estimated rate of missing spots having color *c*. The justification for this rule is given in Appendix 1 ( Supplementary File 1).

We can also update a penalty $\overset{-}{q}$_*c*_ that is associated with *missing* spots of color *c*. This gives a faster way to enforce a desired missing spot rate because there are fewer $\overset{-}{q}$ penalties than *q* penalties. An update to $\overset{-}{q}$_*c*_ is equivalent to a reverse update to all *q _s_* for spots *s* of color *c*, so the update rule is:

$${\overset{-}{q}}_{c}^{\prime }=\frac{\frac{1}{1-p_{fn}(c)}-1}{\frac{1}{P(s)/N}-1}\cdot {\overset{-}{q}}_{c}.\;(2)$$

Typically, we first optimize the $\overset{-}{q}$ parameters to achieve a target missing spot rate, then optimize the *q* parameters to enforce *P*( *s*) ≤ 1 while maintaining the missing spot rate. In either case, we apply Equation 1 or Equation 2 to bring the *q* or $\overset{-}{q}$ parameters close to their final values. When the cost function stops improving, we switch to the steepest-descent algorithms used in Ross and Wiggins, 2012 ^8^ to polish *q* or $\overset{-}{q}$.

**Figure 2.**
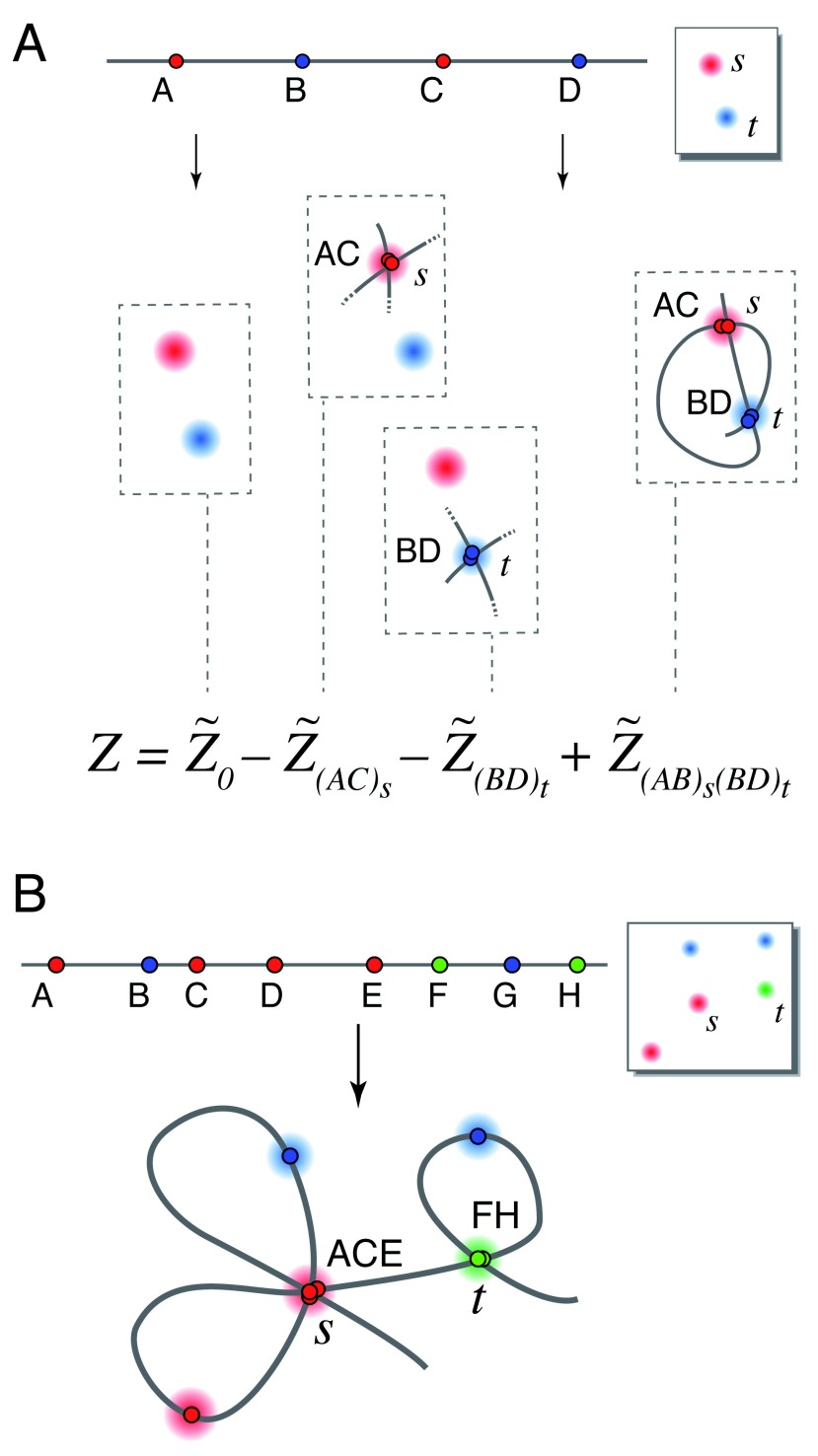
Series expansions. **A**. Schematic showing terms in a series expansion, in a case where series 1 and series 2 have the same terms. The full series gives the exact partition function for the 4-locus experiment shown where only 2 spots appeared in the image (due to a high rate of missing spots). Cartoons show only the constrained loci for each term (so for example each term includes the illegal conformation visiting spots *s* → *t* → *s* → *t*). **B**. An illegal conformation for which loci *A*, *C* and *E* overlap at spots *s*, and loci *F* and *H* overlap at spot *t*. Series expansion 1 includes this conformation in terms ${\tilde{Z}}_{0}$, $\tilde{Z}$_*(ACE)*_*s*__, $\tilde{Z}$_*(FH)*_*t*__, and $\tilde{Z}$_*(ACE)*_*s*_*(FH)*_*t*__. Series expansion 2 includes this conformation in the same terms with the addition of $\tilde{Z}$_*(AC)*_*s*__, $\tilde{Z}$_*(AE)*_*s*__, $\tilde{Z}$_*(CE)*_*s*__, $\tilde{Z}$_*(AC)*_*s*_*(FH)*_*t*__, $\tilde{Z}$_*(AE)*_*s*_*(FH)*_*t*__, and $\tilde{Z}$_*(CE)*_*s*_*(FH)*_*t*__.

Next, we give two exact formulas for the partition functions *Z*_*L*→ *s*_ and the full partition function *Z* that determine our locus-to-spot mapping probabilities. We focus on the full partition function *Z* since the formulas for *Z*_*L*→ *s*_ are identical. The largest term in each formula, which we denote ${\tilde{Z}}_{0}$ (or ${\tilde{Z}}_{0}^{opt}$ when spot penalty optimization is used), is the original estimate from Ross and Wiggins, 2012 ^8^ calculated using a variant of the forward-backward algorithm ^11^. Additional terms are computed in the same way, except that certain loci are constrained to map to certain spots. All of the constraints we will apply are *illegal constraints*, in that they force multiple loci to overlap at some spot in the image; therefore these terms only count illegal conformations that we would like to remove from the baseline calculation. By computing these terms and subtracting them from ${\tilde{Z}}_{0}$ we eliminate the overlapping conformations and improve the calculation. It turns out that this process erroneously subtracts conformations with multiple overlaps more than once and thus we have to add back in higher-order corrections (i.e. partition functions having multiple constrained spots). Repeating this logic leads to exact formulas for *Z* taking the form of series expansions, which are dominated by the lowest-order terms as those have the fewest restrictions on conformational overlaps. Figure 2A illustrates an example of such a series expansion, where each parenthetical subscript ( *X Y* . . . ) _*s*_ on a term label denotes an illegal constraint forcing loci *X*, *Y*, . . . to overlap at spot *s* when that term is calculated. We use this notation throughout.

There are two ways we might remove conformations containing overlapping loci, leading us to two different series expansions for the true partition function *Z*. Suppose that we are calculating the term ${\tilde{Z}}_{{\left(AC...\right)}_{S}}$ whose single illegal constraint forces loci *A*, *C*, . . . to overlap at spot *s*. One option is to forbid any of the other unconstrained loci from also mapping to spot *s*, since spot *s* is already overused. This leads to series expansion 1. Alternatively, allowing further overlaps with spot *s* from the unconstrained loci gives us series expansion 2. Figure 2B illustrates the differences between the two series.

Each of the two series expansions is a weighted sum over *all possible illegally-constrained terms* having two properties: 1) each locus and each spot appear at most once in the indices, and 2) two or more loci map to each constrained spot. To be formal, we use Ω to represent the set of all possible illegal constraints: each element of Ω consists of a set of two or more non-adjacent loci and a single spot where they are forced to overlap. Each expansion thus takes the form

$$Z=\sum_{\phi \subseteq \Omega }w_{\phi }{\tilde{Z}}_{\phi }\;(3)$$

where ${\overset{˜}{Z}}_{\phi }$ is zero if any two constraints share a locus or spot. We will choose the integer weights *w _ϕ_* so as to cancel out the overlapping conformations. By symmetry arguments, the weighting factor should not depend on the identities of the loci or spots, but rather only by the number of constrained spots *n _ϕ_*, and the number of loci $n_{k}^{\phi }$ involved in each *k ^th^* constraint. For example, *w*_( *ACE*) _*s*__ _( *BD*) _*t*__ is determined by *n*_*ϕ*_ = 2, $n_{1}^{\phi }$ = 3 and $n_{2}^{\phi }$ = 2.

Here we specify each series expansion by giving a formula for the weights *w _ϕ_* in terms of *n _ϕ_* and the various $n_{i}^{\phi }$. We also explain how to select an appropriate set of terms *ψ* when there are too many terms to evaluate. Our selection prohibits any legal or overlapping conformation from contributing a negative weight to the partition function estimate, thereby guaranteeing positive mapping probabilities and allowing use of the reconstruction-quality metrics given in Ross and Wiggins, 2012 ^8^. Derivations of the coefficient formulas and the term-selection criteria for each series expansion appear in Appendix 2 ( Supplementary File 1).

**Series expansion 1** For series expansion 1, we do not allow the unconstrained loci to map to spots that were used in constraints. Then the weights *w _ϕ_* in the series formula given by Equation 3 are:

$$w_{\phi }={\left(-1\right)}^{n_{\phi }}\;(4)$$

To select terms for a series approximation, we first choose a set of illegal constraints *ψ* to disallow, then include all series terms ${\overset{˜}{Z}}_{\phi }$ containing only those constraints: i.e. *ϕ* ⊆ *ψ*. This guarantees non-negative mapping probabilities. In order to efficiently evaluate the largest terms, we recommend selecting the *N*_ψ_ constraints having the highest product of mapping probabilities in the baseline calculation ${\tilde{Z}}_{0}$ (or ${\tilde{Z}}_{0}^{opt}$ if spot penalties will be used). For example, we would include ( *AC*) _*s*_ if *p*( *A* → *s*) · *p*( *C* → *s*) is sufficiently large.

**Series expansion 2** For series expansion 2, the unconstrained loci are allowed to map to spots that were used in constraints. Then the weights *w _ϕ_* in Equation 3 are:

$$w_{\phi }=\prod_{k=1}^{n_{\phi }}(-1{)}^{n_{k}^{\phi }-1}(n_{k}^{\phi }-1).\;(5)$$

To select terms for a series approximation, we first choose a set of *N*_ψ_ single-locus-to-spot mappings Ψ, then include all terms *Z _*ϕ*_* whose illegal constraints use only mappings in Ψ. For example, the constraint ( *AC*) _*s*_ would be included if Ψ ⊆ { *A* → *s*, *C* → *s*}. In order to select the largest terms, we recommend building Ψ from the *N*_*ψ*_ largest mapping probabilities calculated from ${\tilde{Z}}_{0}$ or ${\tilde{Z}}_{0}^{opt}$.

## Results

We tested the improved align3d method by generating random chromosome conformations using our software tool wormulator (version 1.1), and simulating the process of error-prone labeling, imaging and finally producing the locus-to-spot mapping probabilities. We considered three scenarios for our simulations. 1) The ‘Toy’ scenario involves 10 genomic loci, where each locus is labeled using one of 3 colors. For these simple problems the partition function can be calculated exactly. 2) Our simulated Experiment 1 uses standard DNA labeling methods and traditional 3-color microscopy to label 30 loci with 3 colors, thus interrogating a significant fraction of a chromosome contour. 3) Our simulated Experiment 2 labels 300 loci across a chromosome-length contour. The reconstruction of Experiment 2 is made possible by using the Oligopaints labeling technique ^4^ to label in 20 different colors.

**Figure 3.**
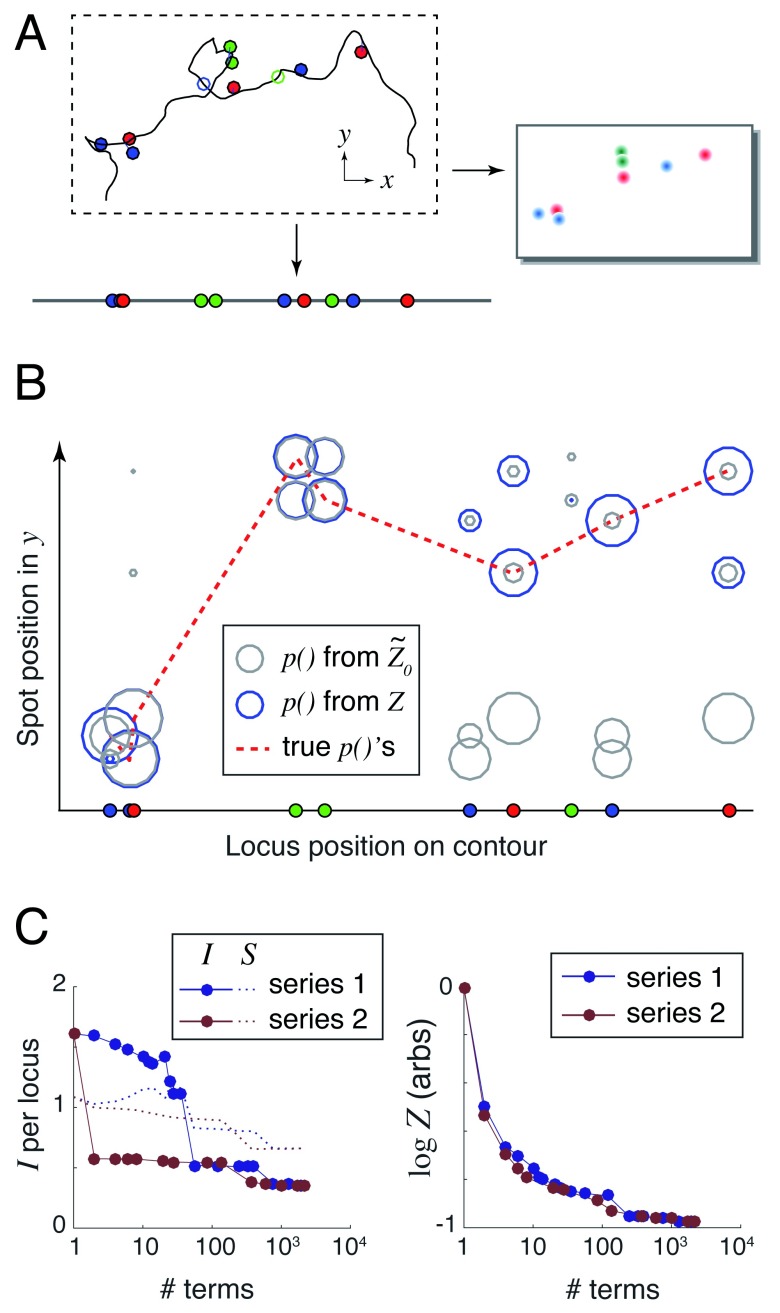
Example reconstruction. **A**. Randomly generated and labeled chromosome contour with simulated experimental error: localization error (lines offsetting spots from the labeled genomic loci) and missing labels (open circles). This example lacks nonspecifically-bound labels (floating spots). **B**. Spot mapping probabilities calculated using both the largest series term ${\tilde{Z}}_{0}$ (grey circles), and the exact *Z* that can be computed using 2210 series terms (blue circles). The dotted red line connects the true locus-to-spot mappings, which are used to calculate the unrecovered information. In this example *I*( ${\tilde{Z}}_{0}$) = 1.54 bits/locus and *I*( *Z* ) = 0.32 bits/locus. **C**. Unrecovered information *I* and entropy *S* (left panel) and log *Z* (right panel) versus the number of terms used in the series expansions.

For each scenario, we randomly generated 100 conformations using a wormlike chain model (packing density *n _l_* = 0.3 kb/nm, persistence length *l _p_* = 300 kb, as suggested by the measurements of Trask, Pinkel and van den Engh, 1989 ^12^); applied a random labeling at a mean density of 1 locus per megabase; and simulated experimental error: 100/200-nm Gaussian localization error in xy/z, a 10% rate of missing labels, and a 10% rate of nonspecifically-bound labels. A typical simulated experiment from the Toy scenario is shown in Figure 3A.

Next, we specified a DNA model relating the genomic distance between two loci *L* to their expected RMS spatial distance *R*, which is used by align3d to estimate the probability density of spatial displacement **r** using a Gaussian chain model: *σ*( **r**) ∝ exp[–3| **r**| ^2^/2 *R* ^2^]. Our current implementation requires a power relation between *R* and *L*, where the exponent may depend on *L*. Since any realistic polymer model predicts straight DNA on very short scales, we chose the model *R* = *n _l_ L* for *L < l _p_* and *R* = *A _*ρ*_ L ^*ρ*^* for *L > l _p_*, where $A_{\rho }=l_{p}^{-(\rho -1)}\cdot n_{l}$ for continuity. In a real experiment the three free parameters *n _l_*, *l _p_* and *ρ* would be fit to pairwise distance distributions between different pairs of loci in a separate calibration experiment. For our purposes *n _l_* and *l _p_* were set to the same values used to generate the wormlike chain conformations, and since these conformations were random walks we set *ρ* = 1/2.

For each simulated conformation, we fed the label positions and colors together with the simulated 3D images and our DNA model into the align3d algorithm to produce locus-to-spot mapping probabilities. For example, the simulated experiment shown in Figure 3A produced the mapping probabilities shown graphically in Figure 3B using circles, where the size of each circle indicates probability magnitude. Here grey circles show the mapping probabilities computed from ${\tilde{Z}}_{0}$ with no use of spot penalties, and blue circles show those same probabilities computed using the exact *Z*. This example shows how excluding high-weight and heavily-overlapping conformations reduces and improves the partition function estimate (see Figure 3C) and concentrates the probability mass into the ‘true’ locus-to-spot mappings (shown connected by the dotted red line in Figure 3B).

Our reconstruction quality metric is the amount of *unrecovered information* from the mapping probabilities, defined as *I* = – 〈log *p*( *L*_i_ → *s*_i_)〉 _i_ where the average 〈.〉 is taken over the set of true locus-to-spot mappings ( *L _i_* , *s _i_*). The ideal case of *I* → 0 implies a perfect reconstruction with no mistakes and zero uncertainty, but in practice *I* is always positive. In a real experiment where the true mappings are not known, we use a proxy for unrecovered information that we term entropy, defined as *S* = − 〈 *p*( *L* *_i_* → *s* *_j_*) log *p*( *L* *_i_* → *s* *_j_*)〉 *_ij_* whose average is taken over all locus-to-spot mappings, not just the correct mappings. The goal is to have *S* ≈ *I* so that a real experiment will have an accurate estimate of the reconstruction performance. The left-hand panel of Figure 3C shows how *I* and *S* depend on the accuracy of the calculation for the simple example shown, using either of the two series expansions and varying the number of terms from 1 (simply ${\tilde{Z}}_{0}$) to 2210 which is the full set of terms for either series and thus computes *Z* exactly. Entropy generally overestimates the amount of unrecovered information (see Supplementary Figure S1 and Supplementary Figure S2, Supplementary File 1), because the large mapping probabilities should be even larger, and the small ones even smaller, than their assigned values (see Supplementary Figure S3, Supplementary File 1). Appendix 3 ( Supplementary File 1) argues that this miscalibration is caused by the mismatch between the wormlike chain DNA model used to generate the simulated conformations and the Gaussian chain model used by align3d in the reconstruction.

**Validation of Equation 1– Equation 5.** We first validated each of the two series expansions by comparing them against exact partition function calculations for the simulated Toy experiments. In all cases, both series expansions, when taken to their maximum number of terms, exactly reproduced the partition function calculations obtained by direct enumeration over all possible non-overlapping conformations. This test validates Equation 4 and Equation 5. We also verified that both series expansions could be used in conjunction with spot penalty optimization ( Equation 1 and Equation 2), both by numerically validating the cost function gradient calculation and by testing for convergence on these small problems.

**Figure 4.**
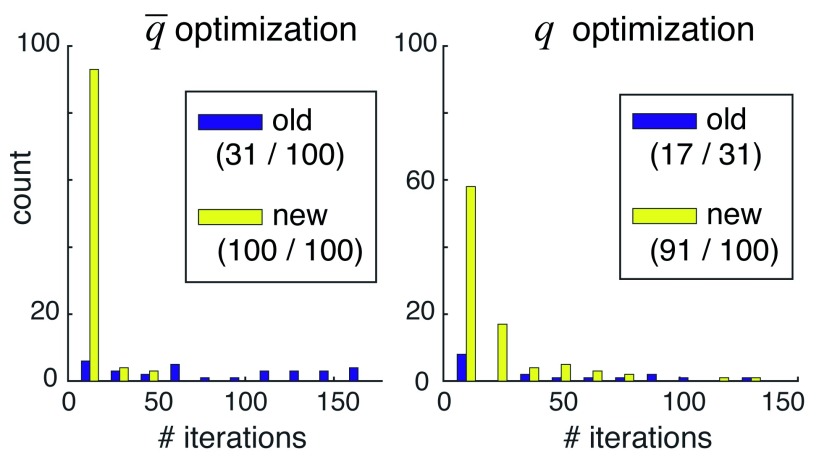
Comparison of old and new optimization methods. Each panel compares the number of iterations required to achieve convergence using the old (purple) versus new (yellow) optimization methods. Only trials that successfully converged are counted, so the histograms are not normalized relative to each other. The first number in parentheses of each legend entry shows the number of converged trials, and the second number shows the total number of trials. Note that the second numbers in the right-hand panel equal the first numbers in the left-hand panel, since we required convergence in $\overset{-}{q}$ in order to attempt optimization of the *q* parameters.

**Improved optimization allows large-scale reconstructions.** Next, we tested whether the iterative spot-penalty optimization rules given by Equation 1 and Equation 2 could work on large-scale problems such as those of Experiment 2, where the old gradient descent optimizer in align3d had difficulty ^8^. The results are shown in Figure 4, which compares the number of iterative steps required to converge the $\overset{-}{q}$ (missing-spot penalty) and *q* (spot penalty) parameters without/with use of our improved optimization rules (labeled ‘old’/‘new’ respectively in the legend). Since the spot penalties *q* are optimized for probability normalization only after $\overset{-}{q}$ parameters have been optimized to achieve a desired missing spot frequency, we only attempted to optimize the *q* parameters for simulations where $\overset{-}{q}$ converged. There were two results from this experiment. First, more attempts to optimize the $\overset{-}{q}$ and *q* parameters successfully converged when using the new optimization rules in conjunction with gradient descent, as indicated by the greater volume of the ‘new’ histogram and the correspondingly larger numbers shown in the legends. Secondly, of the trials that did converge, our new method required significantly fewer iterations and thus less computation time than the old method, as indicated by the relative skews of the distributions.

**Figure 5.**
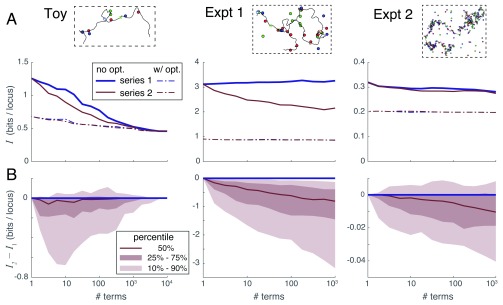
Comparison of the convergence rates of series expansion 1 and series expansion 2. **A**. Median unrecovered information *I* as a function of the number of terms used in each series expansion, without using spot penalty optimization (solid lines) versus with optimization (dotted lines), and over the three simulation scenarios (panels left-to-right). Each curve was derived from the 100 individual curves corresponding to the 100 simulations in each scenario using a simple point-by-point median average. **B**. Percentile distribution of the difference between the unrecovered information using series 2 minus the unrecovered information using series 1; the fact that this difference quickly drops below zero in nearly all individual simulations shows that series 2 recovers more information in the first few terms than does series 1.

**Use of more colors dramatically improves reconstructions.** Our most striking result is that simulations of the ambitious Experiment 2 produce far better results than even the Toy scenario, despite the fact that these simulations have more loci per color than either the Toy scenario or Experiment 1. This can be seen in the amount of unrecovered information *I* shown in the simulation-averaged plots of Figure 5A. High-quality reconstructions using ~ 20 colors were also observed by the ChromoTrace reconstruction method ^9^ even for large numbers of labeled loci. Our explanation is that the reconstruction quality has more to do with the average spatial density of loci per color than the total number of loci per color, because each ‘propagator’ evolving one potential locus-to-spot mapping to the next sees only the spots within some reasonable radius, as determined by the genomic distance to the next locus. These arguments really pertain to the information recovery of the baseline calculation of ${\tilde{Z}}_{0}$; the story is more complicated when better approximating the true *Z* which forbids spot reuse between loci, but a simple heuristic is that some average fraction of the competing spots were used earlier along the contour and should thus removed from consideration. If our reasoning is correct, then reconstructions based on huge numbers of labeled loci (for example whole-genome reconstructions) should be possible as long as the spot density does not get too high.

At the end of this section we revisit Experiment 2, in order to assess the reconstruction quality when analyzing more realistic DNA contours having tighter confinement and thus more closely-packed spots.

**Series expansion 2 outperforms series expansion 1.** Next, we compared the convergence properties of our two expansions on the three scenarios of simulated experiments. Figure 5A gives a sense of how the amount of unrecovered information varies with the number of terms taken in each series, without (solid lines) and with (dotted lines) the use of spot penalties. Each of the 3 panels summarizes all 100 simulated experiments of that scenario, and each experiment in that scenario shows a unique relationship between information recovery and number of series terms computed. Representative curves of individual experiments in each scenario are shown in Supplementary Figure S1 ( Supplementary File 1). In order to summarize these very dissimilar curves, Figure 5A shows a median average of all 100 individual experimental curves taken at each data point. Note that this averaging process does not necessarily preserve the shape of the curves from typical individual simulations.

In order to directly compare the two series expansions, we plotted their difference in unrecovered information *I*_2_ − *I*_1_ versus the number of series terms in Figure 5B. In this case, we plotted the full distribution showing the median (50th percentile) as well as the 10th, 25th, 75th and 90th percentile curves. These plots show directly that series 2 almost always outperforms series 1 when only a few terms can be evaluated. The reason is that the terms in series 2 are larger in magnitude owing to their looser constraints, and thus remove the extraneous part of the partition function more quickly than the terms of series 1 (see Supplementary Figure S1 and Supplementary Figure S4, Supplementary File 1). Based on these results, we recommend using series expansion 2 in all situations where the partition function cannot be evaluated exactly.

**Figure 6.**
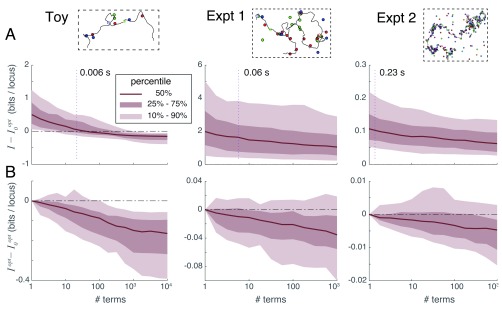
Optimization in conjunction with series expansions. **A**. Comparison of unrecovered information using series expansions without iteration, denoted *I*, to the unrecovered information obtained by optimizing spot penalties using only the first series term, denoted $I_{0}^{opt}$, over three experimental situations. Vertical dotted lines indicate the median number of series terms computable with the same computational time as was required to obtain $I_{0}^{opt}$. For Experiments 1 and 2 the difference $I-I_{0}^{opt}$ is typically positive at the intersection of the dotted line, indicating that spot penalty optimization method is the more efficient way of recovering information. **B**. Comparison of unrecovered information using spot-penalty optimization in conjunction with multiple series terms versus optimization of ${\tilde{Z}}_{0}$ alone, showing the added benefit of including more terms in the series.

**Spot penalty optimization is the most efficient way to recover information.** Spot penalty optimization is an iterative process where each iterative step requires the evaluation of some number of series terms. An optimization requiring *t* iterations thus multiplies computation time by a factor of *t* relative to the simple evaluation of the series. Alternatively, one could spend the extra computation time on taking the series to a higher order without spot penalty optimization. Figure 6A plots the unrecovered information when a) taking series 2 to a certain order without optimization, versus b) using spot penalty optimization on only the first term yielding ${\tilde{Z}}_{0}^{opt}$ . The dotted line in each panel shows the median number of terms requiring the same computation time as ${\tilde{Z}}_{0}^{opt}$ . The Toy scenario shows that, if the series expansion is carried deep enough, it becomes more accurate than ${\tilde{Z}}_{0}^{opt}$ : in other words the difference $I-I_{0}^{opt}$ becomes negative. However, for the practical scenarios of Experiments 1 and 2 this crossover point requires taking more terms than would be needed to match the computational cost of calculating ${\tilde{Z}}_{0}^{opt}$ (the dotted line). Based on these results, we recommend always performing spot penalty optimization, especially for larger reconstructions.

**Series expansions can improve optimization information recovery.** Although spot penalty optimization is the most efficient way to recover information, that process alone can only extract a certain fraction of the recoverable information: once the cost function is zero, optimization can proceed no further despite the problem not having been solved exactly. At this point, the only way forward is to go higher in the order of series terms used; we can still apply spot penalties to this sum of terms and iteratively optimize them as before using Equation 1 and Equation 2. Figure 6B plots the difference in unrecovered information when applying spot penalty optimization between a) a variable number of terms in series expansion 2, and b) only ${\tilde{Z}}_{0}$ (the first series term). This figure shows that including additional series terms in the optimization improves the information recovery, albeit at a slow rate (especially for large problems).

**20-color labeling leads to near-perfect reconstructions.** As shown in Figure 5A, the unrecovered information for the whole-chromosome Experiment 2 averages around 0.2 bits per locus, implying near perfect mapping probabilities. However, because these results were based on randomly-generated unconfined conformations, they do not establish whether such good information recovery is possible with real chromosomes which are likely to be more compact. To test Experiment 2 on realistic chromosome conformations, we generated four plausible conformations of human chromosome 4 by running the GEM software package ^13^ on the smoothed human Hi-C data set provided by Yaffe and Tanay, 2011 ^14^ and using a 3D spline interpolation to increase the resolution from 1 Mb to 50 kb. These conformations were then virtually labeled at 300 randomly-selected loci and simulated experimental error was added in as before. One set of experiments assumed diffraction-limited 100/200 nm localization error in xy/z, and a second set of experiments assumed superresolution 30/50 nm localization error in xy/z; in both sets the missing- and extra-spot rates were 10%. For this experiment we determined the DNA model parameters *n _l_* and *l _p_* by fitting pairwise locus distributions, as one would do in an experiment, and for *L > l _p_* we set *ρ* = 1/3 as that has been reported in the literature for locus separations under 7 Mb ^4^. Mapping probabilities were reconstructed by taking series expansion 2 to the lowest order that included at least 1000 terms, then applying and optimizing spot penalties. Compared with the random-walk conformations used to test the Experiment 2 scenario, the diffraction-limited reconstructions did somewhat worse (~ 0.4 versus ~ 0.2 bits of unrecovered information per locus) owing to fact that physical confinement of chromosomes increases the density of competing spots in the image. The superresolution reconstruction quality was unchanged at ~ 0.2 bits of unrecovered information.

**Figure 7.**
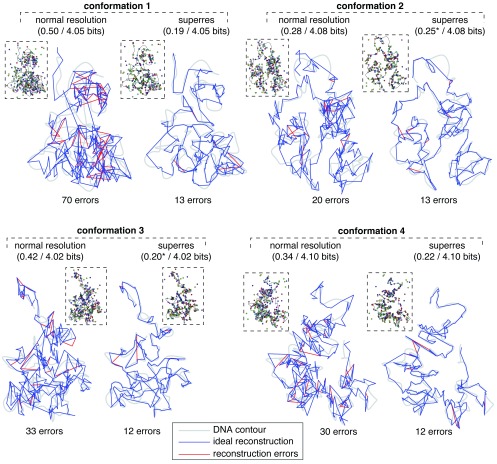
Simulated reconstructions of 4 plausible conformations of human chromosome 4. The left-hand reconstruction of each conformation was obtained using a simulated image from diffraction-limited microscopy (shown in inset; localization error is shown as lines connecting spots to DNA), and the right-hand reconstruction used a simulated superresolution image. Grey shaded lines indicate the underlying DNA contours; blue lines trace the ideal reconstructed contours given the measured spot positions; red lines show our reconstructed contours where they deviate from the ideal contours. Captions above each reconstruction indicate the amount of unrecovered information *I* per locus after/before the reconstruction process; captions below indicate the number of alignment errors between the spot ID sequences read along the true versus inferred conformations. For both superresolution reconstructions 2 and 3 we calculated *I* excluding a single locus whose true spot mapping was given 0 probability; including that locus sends *I* → ∞.

Despite the drop in performance when localizing spots at the diffraction limit, 0.4 bits of unrecovered information per locus is still an extremely strong reconstruction, implying that the correct locus-to-spot mappings are assigned *p*-values averaging around 2 ^–0.4^ ≈ 76%. Starting from such accurate and confident mapping probabilities, one can infer a reasonable conformation simply by assigning each locus to the unassigned spot to which it maps with the highest probability (or calling a missing spot if $1-\sum_{s\prime }\;p_{L\to s^{\prime }}$ > any *p*_*L*→ *s*_), repeating the process for overlapping loci, and drawing a line in the image that connects these spots in genomic order. The conformations produced by this simple rule are shown in Figure 7: the correct conformation is shown with a blue line and errors in the inferred conformation are shown in red. The reconstructed conformations are ~ 90% accurate at diffraction-limited resolution and ~ 96% accurate at superresolution, as determined by an alignment between the true and inferred spot sequences traveling along the DNA contour. Most mistakes are of a sort that does not change the large-scale structure. For example, one common error is to erroneously skip one or more spots in the image, thus ‘looping out’ a small part of the conformation and effectively lowering the resolution.

Figure 7 shows that the benefit of superresolution is twofold: 1) the locus-to-spot mapping quality improves relative to diffraction-limited resolution (i.e. fewer red lines), and 2) the small-scale structure of an ideal mapping (blue line) more faithfully traces the underlying contour (grey line). This shows the importance of measuring spot locations to sub-pixel resolution, even in experiments where normal-resolution microscopes using standard fluorophores are used to localize spots separated by two pixels or more. In our GEM conformations 23 spots were closer than 200 nm to another spot of the same color, which would indicate problems localizing these spots, but this is inconsistent with the data shown in Wang *et al*., 2016 ^4^ which indicates that virtually all spots in our experimental scenarios should be well-separated in at least in some cell lines.

## Discussion

We have developed and evaluated two improvements to the align3d method for reconstructing chromosome structure. Both of these improve the partition function estimates that determine the locus-to-spot mapping probabilities, which can provide the basis for (probabilistic) reconstructed conformations. The first improvement is a more robust spot-penalty optimizer that allows for large-scale reconstructions involving hundreds of labeled loci, such as will be needed to uncover whole-chromosome conformations. The second improvement is two series expansion formulas for the partition functions, which in principle allow the mapping probabilities to be solved to arbitrary accuracy within the limitations of the experiment and the underlying DNA model. In practice, the series approach is difficult for two reasons: 1) there are a huge number of terms in each series expansion, and 2) the lowest-order approximation ${\tilde{Z}}_{0}$ overestimates *Z* by many orders of magnitude, unlike other series expansions where the initial approximation is close to the final answer. Despite the difficulties, the series formulas that we give offer some way forward to improve on the original estimate ${\tilde{Z}}_{0}^{opt}$. Of the two formulas, we recommend using series expansion 2, which has the larger-magnitude terms and thus recovers the most information when only a few terms can be evaluated.

Our problem of finding likely (i.e. low-free-energy) DNA conformations passing through a set of imaged spots is similar to the well-known traveling salesman problem (TSP), in which a salesman must find the shortest route connecting a set of cities. Somewhat more closely related is a generalization of the TSP called the time-dependent traveling salesman problem (TDTSP) ^10^, where the intercity distances change every step on the tour; this is analogous to our situation where the free energy needed to thread DNA between two spots depends not only on their separation but also on the length of DNA used to connect them. In our case, the presence of missing and extra spots generalizes our problem still further: in the TDTSP analogy the salesman would be allowed to skip stops and cities for a penalty. Our main finding is that the partition function of this generalized TDTSP (which encompasses traditional TSP and TDTSP problems) can be expressed as a sum of terms computable using a (modified) forward-backward algorithm, a result which should also apply to other route-finding applications where research has historically focused on route optimization rather than route inference.

Both our mapping *p*-values and our entropy proxy for information recovery show a systematic bias, which comes from the use of a different DNA model for reconstruction than was used to create the simulated DNA contours. The fact that our reconstructions were nonetheless quite strong shows that the reconstruction method itself is quite robust to model error. This is very fortunate given the uncertainty in the true *in vivo* biological model describing the cells in a real experiment. For our results to be accurate, we had to calibrate our model so as to reproduce the peak in the distance distribution of pairs of distinguishable loci. An experimenter would perform this calibration by imaging distinguishable pairs of loci in a parallel experiment. Due to align3d’s use of a very permissive Gaussian chain DNA model, both systematic biases work in the direction of causing the method to underestimate its performance: high *p*-values should be higher (and low *p*-values lower) than reported, and the unrecovered information tends to be less than the entropy estimate. Thus the results are at least as good as they appear to be.

From a genomic standpoint, our most exciting result is that the combination of our computational improvements together with 20-color labeling technology gives almost perfect reconstructions at the whole-chromosome scale. Out of ~ 4 bits per locus of uncertainty inherent in the reconstruction problem, our method recovers ~ 3.6–3.8 bits. Such confident mapping probabilities allow for the direct construction of individual conformations that are ≥ 90% accurate. High-quality piecewise reconstructions are likewise possible with two overlapping copies of the same chromosome (data not shown), although sometimes the fragments cannot be assembled. We want to emphasize that our reconstructions require only a few parameters that would be known experimentally with proper controls: the 3 DNA model parameters which in a real scenario would be calibrated using a control experiment, and the correct average rates of missing and extra spots averaged over all experiments, used by align3d to estimate the actual number of missing spots per color in each experiment. The robustness of the analysis to experimental unknowns gives evidence that reconstructions using real-world experimental data will be of similar quality to those in our simulations, and if so then direct measurement of chromosome conformations is possible today with current technology.

## Data availability

The simulated conformations and labelings used to generate the plots in this paper, together with the output of the align3d analysis, can be found at: https://github.com/heltilda/align3d/blob/master/seriesExpansions/a3dRawData.zip

## Software availability

Results in this paper were generated using version 1.1 of align3d, built using version 1.1 of Cicada scripting language. Simulated conformations and labelings were generated using version 1.1 of wormulator.

All source files used in preparing this paper are available from the GitHub page for this paper: https://github.com/heltilda/align3d/tree/master/seriesExpansions.

License: GPL 3.0

Archived code at time of publication:

align3d: https://doi.org/10.5281/zenodo.2580342 ^15^

License: GPL 3.0

wormulator: https://doi.org/10.5281/zenodo.1411503 ^16^

License: GPL 3.0

Cicada scripting language: https://doi.org/10.5281/zenodo.1411505 ^17^

License: MIT License

## Footnotes

1 Depending on how the experiment is done, two spots of the same color sufficiently close in the image may appear as a single spot where the conformation self-overlaps. We prefer to treat this scenario as a missing-spot measurement error rather than relax the one-spot-per-locus rule. If the spots have been properly localized, then the underlying conformation visits any given spot once at most.

## Supplementary material

Supplementary File 1: align3dSupplement.pdf. File containing three appendices giving the derivations of the equations used in this text (Appendix 1 and Appendix 2), a discussion of model error (Appendix 3), and the supplemental figures (Appendix 4).
