# Supplementary material for: Improved inference of chromosome conformation from images of labeled loci
Source: F1000Res. 2019 Mar 28;7:ISCB Comm J-1521. Originally published 2018 Sep 21. [Version 3] doi: 10.12688/f1000research.16252.3 (PMC6644830; doi:10.12688/f1000research.16252.3)
Supplement: Supplementary file 1 [file f1000research-7-20533-s0000.tgz › bdc6b253-d39b-459d-b1f8-91ce179623ee_Supplementary_file_1_v2.pdf]

Supplementary Material

Appendix 1: justification for gradient-free weight update rule

The optimization of the spot penalties uses a cost function that is entirely determined by the total probability $P(s)$ of mapping a locus to a given spot s in the image:

$$P(s) = \sum_L p_{L\to s} = \frac{1}{Z}\sum_L Z_{L\to s}.$$

Both $Z_{L\to s}$ and Z sum terms that are products of the spot penalties q_s, owing to the fact that a direct influence on any one mapping probability indirectly influences all other mapping probabilities. However, when the penalty on some spot s is far away from its proper value, any mapping probability $p_{L\to s}$ to that spot tends to be saturated very close to either 0 or 1. In this case we make the approximation that a given penalty factor q_s only affects any given $p_{L\to s}$ directly as a multiplying factor, and does not affect the mapping probabilities to other spots, since the indirect influences are small until the mapping probability is out of saturation. Under this approximation $Z_{L\to s} \approx f_s \cdot a_s$, and $NZ = \sum_L \sum_s Z_{L\to s} \approx f_s \cdot a_s + b_s$, so

$$P(s) \approx \sum_L \frac{f_s N}{f_s + (b_s/a_s)}. \tag{6}$$

Since our objective is to find an updated q'_s causing the sum of mapping probabilities to be some target $P'(s)$, we also write:

$$P'(s) \approx \sum_L \frac{f'_s N}{f_s + (b_s/a_s)}. \tag{7}$$

Solving Eqs 6 and 7 together to eliminate the unknown b_s/a_s gives us the update rule:

$$f'_s \approx \frac{\frac{1}{P(s)/N} - 1}{\frac{1}{P'(s)/N} - 1} \cdot f_s.$$

The cost function contains two terms: 1) a penalty for the overall difference between the expected rate of missing spots p_{fn} versus that inferred from the summed $P(s)$; and 2) a penalty on $P(s)$ if it exceeds 1, which would indicate a $>100\%$ likelihood of a locus mapping to that spot. Our current implementation simply makes two updates, one pushing $P(s) \to p_{fn}$ and the second pushing $P(s) \to 1$ for spots violating normalization. Doing so leads to the update rule given by Equation 1.

Appendix 2: series expansion derivations

Here we prove a) each series converges to the true partition function when all terms are counted, and that b) a given truncation of each series using our recommended selection of series terms counts every possible legal or overlapping conformation zero or more times (despite many series terms having negative coefficients), and therefore produces positive mapping probabilities. Notice that both expansions count all legal (non-overlapping) conformations once from $\tilde{Z}_0$, and the goal of considering higher-order series terms is thus to minimize the weight of the illegal overlapping conformations without their weights ever becoming negative. We note that conformations overlapping at adjacent loci are automatically eliminated from all terms in the calculation, so the individual conformations we consider here are assumed to overlap themselves only between non-adjacent loci.

For our proofs we will define an illegal overlap as a set of loci in an illegal conformation mapping to a given spot in the image. Illegal overlaps are to illegal conformations as illegal constraints are to higher-order series terms, and we use the same set notation for overlaps as we do for constraints.

Our derivations make repeated use of the following identity, taken from Equation 3.1.7 in Ref. [15].

$$\sum_{b=0}^{a} \binom{a}{b} (-1)^b = 0 \text{ for } a > 0 \tag{8}$$

This relation follows from the fact that the left-hand side is a series expansion for $(1-1)^a$.

Series expansion 1 A given conformation bearing a set of illegal overlaps θ is counted by each constrained partition function whose set of illegal constraints (indices) are a subset of θ. Thus the weight W_θ of this conformation in the full partition function Z is:

$$W_\theta = \sum_{\phi\subseteq\theta} w_\phi = \sum_{n_\phi=0}^{n_\theta} \binom{n_\theta}{n_\phi} (-1)^{n_\phi} = \begin{cases} 1 \text{ for } n_\theta = 0 \\ 0 \text{ otherwise} \end{cases}$$

where we have used Equation 8 in the final step. This result shows that series expansion 1 counts only conformations having no overlaps, i.e. for which $n_\theta = 0$.

Suppose that one follows our prescription for evaluating a subset of terms, namely all terms that are a subset of overlaps ψ. Then using the same formula, we reason that a conformation with overlaps θ will be given the following weight:

$$W_\theta^{(\psi)} = \sum_{\phi \subseteq (\theta \cap \psi)} w_\phi = \begin{cases} 1 \text{ for } \theta \cap \phi = \emptyset \\ 0 \text{ otherwise.} \end{cases}$$

Thus our selection of series terms for expansion 1 eliminates all conformations from the partition function having any overlaps contained in our set ψ.

Series expansion 2 Expansion 2 allows unconstrained loci to revisit spots that already have constrained loci. In this case, a conformation whose set of nonadjacent overlaps is denoted θ will be counted by a partition function having overlaps ϕ if each individual overlap ϕ_i is *contained in* an individual overlap θ_i, in the sense that ϕ_i maps any subset of $n_i^\phi > 1$ loci in θ_i to the same spot as θ_i. Then the weight of this conformation in the expansion is

$$\begin{aligned} W_\theta &= \sum_{\phi = \{\phi_i \subseteq \theta_i\}} w_\phi \\ &= \prod_{k=1}^{n_\theta} \left[1 + \sum_{n_k^\phi = 2}^{n_k^\theta} \binom{n_k^\theta}{n_k^\phi} (-1)^{n_k^\phi - 1} (n_k^\phi - 1) \right] \\ &= \prod_{k=1}^{n_\theta} \sum_{n_k^\phi = 0}^{n_k^\theta} \binom{n_k^\theta}{n_k^\phi} (-1)^{n_k^\phi - 1} (n_k^\phi - 1) \\ &= \prod_{k=1}^{n_\theta} \sum_{n_k^\phi = 1}^{n_k^\theta} n_k^\theta \binom{n_k^\theta - 1}{n_k^\phi - 1} (-1)^{n_k^\phi - 1} \end{aligned}$$

where the fact that $n_k^\theta > 0$ allowed Equation 8 to eliminate a term in the last line. Defining $m_k^\phi = n_k^\phi - 1$ gives us:

$$W_\theta = \prod_{k=1}^{n_\theta} n_k^\theta \sum_{m_k^\phi = 0}^{n_k^\theta - 1} \binom{n_k^\theta - 1}{m_k^\phi} (-1)^{m_k^\phi} = \begin{cases} 1 \text{ for } n_\theta = 0 \\ 0 \text{ otherwise.} \end{cases}$$

again using Equation 8. Therefore the complete series counts only non-overlapping conformations.

Our prescription for choosing series terms is to select a set of single-locus-to-spot mappings Ψ, and generate all series terms whose illegal overlaps are built only from mappings in Ψ. To be formal, we will compile all locus-to-spot-s-mappings in Ψ for each given spot s (assuming there are 2 or more) into a single overlap, and use ψ to denote the set of these overlaps: then the series order N_ψ is the sum of the number of loci over all elements ψ_i. Our rule is to include all series terms ϕ whose illegal overlaps are built entirely from *subsets of* ψ, in the sense that a given overlap to spot s contains a subset of the loci in the element ψ_i that maps to spot s. For example if ψ contains the element $(ACE)_s$ then $\phi \supseteq \{(AC)_s, (CE)_s, (AE)_s, (ACE)_s\}$. Using these rules, a given conformation having illegal overlaps θ will be given the following weight in the expansion:

$$\begin{aligned} W_\theta^{(\psi)} &= \sum_{\phi = \{\phi_i \subseteq (\theta_i \cap \psi_i)\}} w_\phi \\ &= \prod_{k=1}^{n_{\theta\cap\psi}} \sum_{n_k^\phi = 0}^{n_k^{\theta\cap\psi}} \binom{n_k^{\theta\cap\psi}}{n_k^\phi} (-1)^{n_k^\phi - 1} (n_k^\phi - 1) \\ &= \begin{cases} 1 \text{ if all } n_k^{\theta\cap\psi} < 2 \\ 0 \text{ otherwise.} \end{cases} \end{aligned}$$

Thus series 2 eliminates all conformations having any overlap $(L_1, L_2, \dots) \to s$ where $L_1 \to s$ and $L_2 \to s$ are contained in Ψ.

In some instances we might want to enforce a set of *legal* constraints γ on the calculation of the mapping p-values, in order to test what happens to the rest of the mapping under those assumptions. For example if we set $\gamma = \{L_1 \to s_1\}$ which forces locus L_1 to map to spot s_1, then $Z_{(L_2 \to s_2)\gamma}/Z_\gamma$ calculates the *conditional* likelihood of finding mapping $L_2 \to s_2$ under the assumption that $L_1 \to s_1$. In order to calculate the conditional full and mapping partition functions Z_γ and $Z_{(L_2 \to s_2)\gamma}$, we simply remove the set of legally-mapped loci and spots from consideration and apply series formula 4 or 5 to the set of unmapped loci and spots. Viewed another way, we simply remove every term $Z_{\gamma \bigcup \theta}$ where θ contains loci or spots found in γ from both expansions. To be consistent, we then forbid *unconstrained* loci from mapping to legally-constrained spots contained in γ, even when computing a term in series 2 which does allow them to map to illegally-constrained spots contained in θ.

Appendix 3: model error

In any real experiment there will be some discrepancy between the underlying DNA model and the Gaussian chain model used in the reconstruction. This discrepancy was also captured in our simulated experiments, whose DNA contours were generated using a wormlike chain model, not a Gaussian chain. Here we show that model error causes our program's output to underestimate the reconstruction accuracy in two respects: 1) the reported mapping p-values are systematically shifted towards the mean with respect to their true likelihood of being a true mapping (as we see in Figure S3), and 2) as a result the entropy of the mapping probabilities S tends to overestimate the true amount of unrecovered information I in a reconstruction experiment.

The mapping p-values are estimated by summing the statistical weights of individual conformations, which are each proportional to the product of statistical weights for stretching each adjacent pair of mapped loci (L_i, L_j) between their respective spots (s_i, s_j). Note that $j = i+1$ unless there are unmapped loci (i.e. missing spots) in between. The weight assigned to a given conformation is:

$$Z_{conf} \propto \exp\left[-\sum_{L_i} F(L_i, s_i, L_j, s_j)\right]$$

where $F(L_i, s_i, L_j, s_j)$ is the free energy required to connect two spots mapped by loci L_i and L_j, as determined by the DNA model. We require that our model be correctly calibrated in the sense that the estimated free energy $F(L_i, s_i, L_j, s_j)$ correlates positively with the true free energy $\bar{F}(L_i, s_i, L_j, s_j)$ by a factor $\lambda \leq 1$. Roughly speaking, this requires that 1) the minimum-free-energy peak of our inferred inter-locus distance distribution match the peak of the true distribution (which can be measured in calibration experiments), and 2) that our approximate model should decay away from that peak as slowly or more slowly than the true distribution does. This latter requirement justifies our use of a Gaussian chain model for reconstruction, as that distribution decays much more gradually off-peak than realistic polymer distributions. If these requirements are met, the effect of model error is to partially de-correlate the free energy estimates from their true values, which we model by writing $F = \lambda\bar{F} + R$ where R is a random term.

Next we assume that the random term R is uncorrelated with whether two loci considered by our calculation are actually connected in the underlying true conformation. In other words, we assume that there is no conspiracy between the discrepancies in the DNA model for real versus competing spots mapped to loci i and j, whether the competing spots come from other locations on the DNA contour or are extraneous or nonspecifically-bound labels. Under this assumption, the weight Z_κ assigned to any inferred (real or incorrect) conformation having locus-to-spot mappings κ is proportional to:

$$Z_\kappa \propto \exp\left[-\sum_{L_i}\left(\lambda_i \bar{F}(L_i, s_i, L_j, s_j) + R_i\right)\right].$$

Assuming that the DNA contour spans many loci, the variance in the random terms between conformations is much less than the variance in $\bar{F}$. In the limit where the random sum is constant the conformational weight is:

$$\begin{aligned} Z_\kappa &\propto \exp\left[-\sum_{L_i}\left(\lambda_i \bar{F}(L_i, s_i, L_j, s_j)\right)\right] \\ &= \bar{Z}_\kappa^\lambda \end{aligned}$$

where $\lambda \leq 1$ is some average of the λ_i, and $\bar{Z}_\kappa$ is the true statistical weight of the conformation, i.e. the weight that would be assigned by the correct DNA model. Thus the effect of model error is to reduce the variance in the conformational weights, by a constant exponent in our simple approximation.

Next we consider the effect of model error on the mapping probabilities $p_{L\to s}$, where s can denote either a spot in the image or a missing spot at position L.

$$\begin{aligned} p_{L\to s} &= \frac{Z_{L\to s}}{Z} \\ &= \frac{\sum_{\kappa\in\{L\to s\}} \bar{Z}_\kappa^\lambda}{\sum_\kappa \bar{Z}_\kappa^\lambda} \end{aligned}$$

In the limit where $\lambda = 1$, i.e. a perfect model, we have $p_{L\to s} \to \bar{p}_{L\to s}$, i.e. the true p-values given the uncertainty in the data. In the limit where $\lambda = 0$, we have $p_{L\to s} \to p_{0_L}$, where p_{0_L} is the *unweighted* fraction of conformations for which $L \to s$ and can be thought of as some 'average' p-value for that locus. For intermediate values of the model error, we find that the p-values go monotonically between these two extremes by looking at how they vary with λ:

$$\begin{aligned} \frac{dp_{L\to s}}{d\lambda} &= \frac{\left(\sum_{\kappa\in\{L\to s\}} \bar{Z}_\kappa^\lambda \log \bar{Z}_\kappa\right)\left(\sum_\kappa \bar{Z}_\kappa^\lambda\right)}{\left(\sum_\kappa \bar{Z}_\kappa^\lambda\right)^2} \\ &\quad - \frac{\left(\sum_{\kappa\in\{L\to s\}} \bar{Z}_\kappa^\lambda\right)\left(\sum_\kappa \bar{Z}_\kappa^\lambda \log \bar{Z}_\kappa\right)}{\left(\sum_\kappa \bar{Z}_\kappa^\lambda\right)^2} \\ &= \frac{\sum_{\kappa_1\in\{L\to s\},\kappa_2} \bar{Z}_{\kappa_1}^\lambda \bar{Z}_{\kappa_2}^\lambda \log\left(\bar{Z}_{\kappa_1}/\bar{Z}_{\kappa_2}\right)}{\left(\sum_\kappa \bar{Z}_\kappa^\lambda\right)^2} \end{aligned}$$

This is the mean of $\log\left(\bar{Z}_{\kappa_1}/\bar{Z}_{\kappa_2}\right)$ taken over all pairs of mapped/unmapped conformations κ_1 and κ_2, weighted by their estimated joint likelihood. Thus if κ_1 conformations having the mapping $L \to s$ have higher statistical weight than a typical conformation κ_2, then the derivative is uniformly positive, so that the probabilities monotonically increase as the model becomes more accurate $(\lambda \to 1)$. If κ_1 conformations have lower statistical weight than a typical conformation, then $\log\left(\bar{Z}_{\kappa_1}/\bar{Z}_{\kappa_2}\right) < 0$, so that the probabilities monotonically decrease as the model becomes more accurate. Thus the presence of model error causes the assigned mapping probabilities of putative mappings $L \to s$ to be less extreme than they should be, and increasing amounts of model error increase the tendency for these p-values to move towards the mean. To summarize this we write $p_{L\to s} = \mu\bar{p}_{L\to s} + (1-\mu)p_{0_L}$, where the new parameter μ monotonically increases with λ and has the same limits: $\mu = \lambda = 0$ for a very poor DNA model, and $\mu = \lambda = 1$ for a perfect DNA model.

Finally, we consider the effect of model error on the error in the entropy estimate S, which attempts to measure the amount of unrecovered information I. Specifically, we look at the quantity $S - \langle I \rangle$, which is the expected error in using entropy as an information estimate averaged over the various possibilities for the unknown true conformation $\bar{\kappa}$.

$$
\begin{aligned}
S - \langle I \rangle &= -\sum_{L,s} p_{L\to s} \log p_{L\to s} + \sum_{\bar{\kappa}} \bar{p}_{\bar{\kappa}} \sum_{L} \log p_{L\to s_{\bar{\kappa},L}} \\
&\approx -\sum_{L,s} (p_{L\to s} - \bar{p}_{L\to s}) \log p_{L\to s}
\end{aligned}
$$

Here $\bar{p}_{L\to s}$ is the actual likelihood of the underlying conformation having the mapping $L \to s$, given the unknown true DNA model. Accounting for model error gives us:

$$
\begin{aligned}
S - \langle I \rangle &= -\sum_{L,s} ((\mu \bar{p}_{L\to s} + (1-\mu) p_{0_L}) - \bar{p}_{L\to s}) \log p_{L\to s} \\
&= (1-\mu) \sum_{L,s} (p_{0_L} - \bar{p}_{L\to s}) (-\log p_{L\to s}).
\end{aligned}
$$

Next we look at the sign of the sum. Without the $\log p$ weighting factor, the sum over spots for fixed L would be $\sum_s (p_{0_L} - \bar{p}_{L\to s}) = 1 - 1 = 0$. However, the factor $(-\log p_{L\to s})$ is always positive, and largest for the smallest p-values, i.e. those for which $p_{0_L} - \bar{p}_{L\to s}$ is most positive. Thus the overall sum over $(p_{0_L} - \bar{p}_{L\to s}) (-\log p_{L\to s})$ is positive whenever there is any variability in the mapping probabilities. This result shows that $S - \langle I \rangle > 0$ in the presence of model error, and increases linearly with decreasing μ.

Appendix 4: Supplementary figures

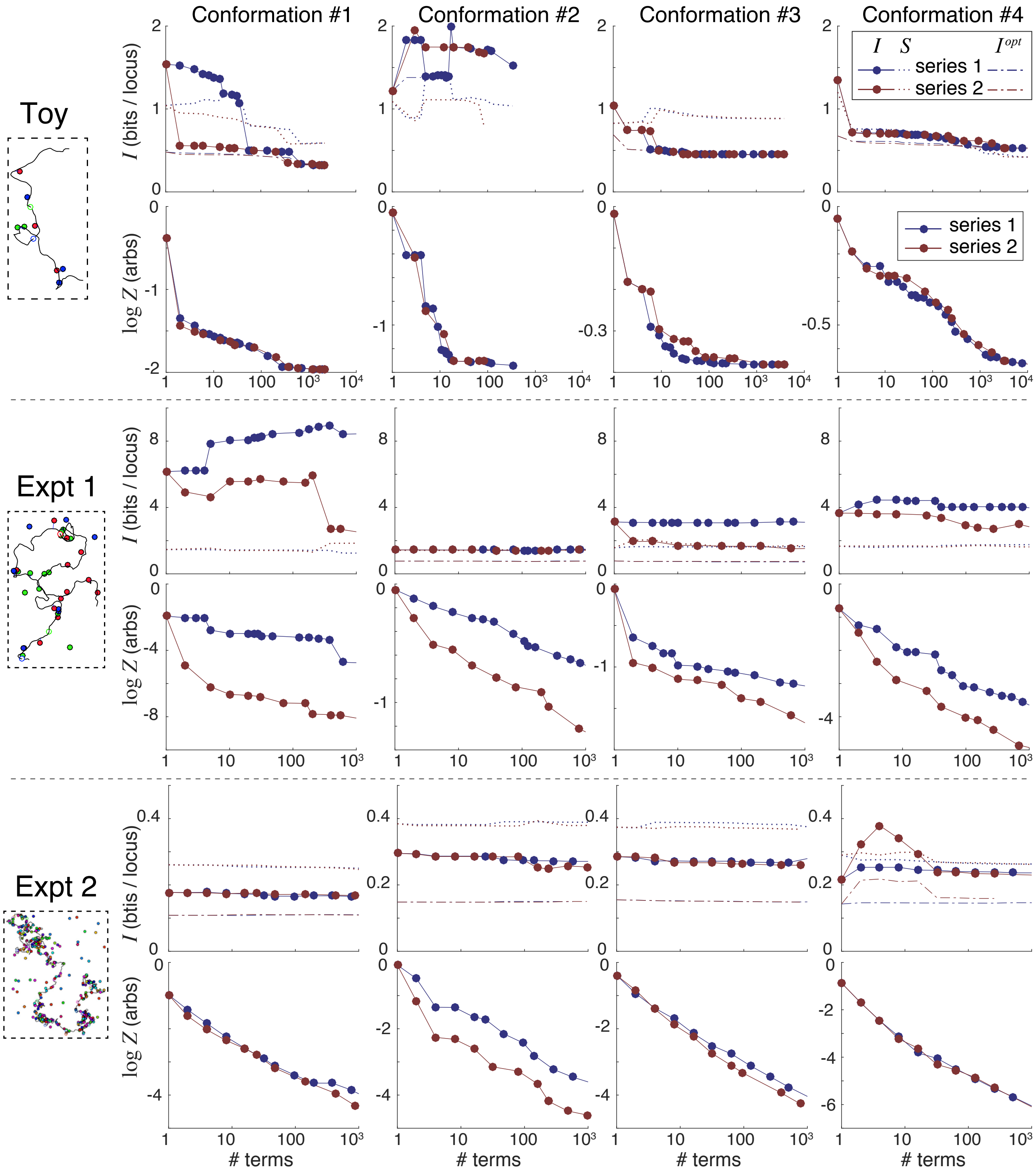

Figure S1. Information recovery from individual simulations. Conformation #1 of each series is shown at far left. Panels to the right show unrecovered information (I), entropy (S) and $\log Z$ as a function of the number of series terms included. Dot-dashed lines show the unrecovered information of $\tilde{Z}^{opt}$ using optimized spot penalties together with the given set of the series terms. Entropy of $\tilde{Z}^{opt}$ is not shown.

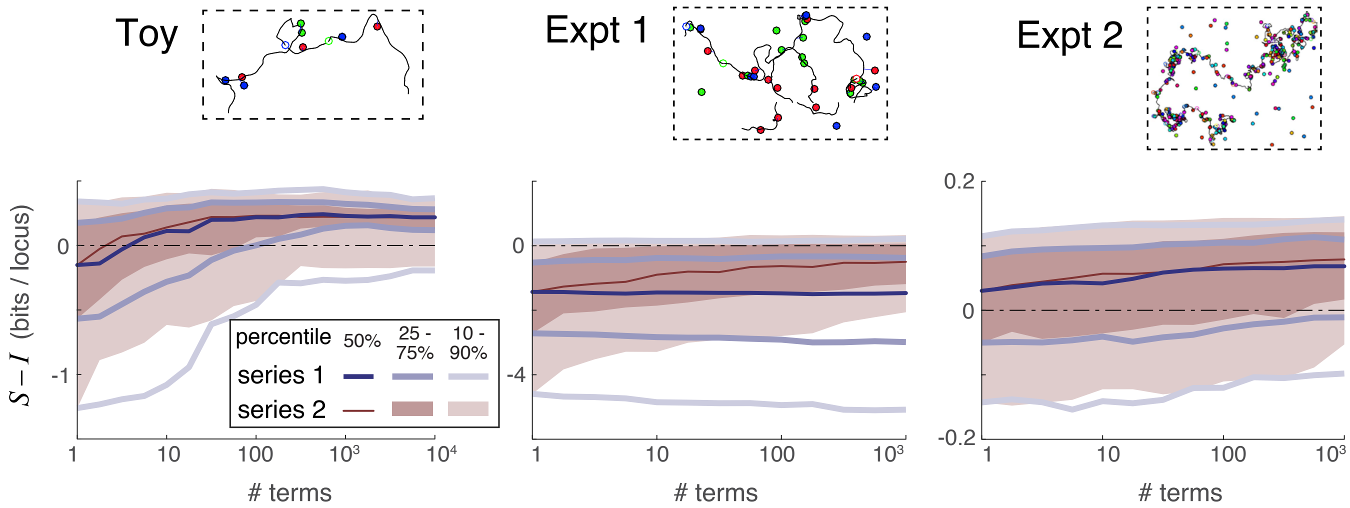

Figure S2. Accuracy of entropy as a proxy for unrecovered information. Distributions showing the difference between entropy S, which is a blind estimate of unrecovered information, and actual unrecovered information I in each of the 3 simulation scenarios considered, as a function of number of series terms. No spot penalties were used for these results. Each distribution shown encompasses the $S - I$ curves of all 100 simulated reconstructions in one scenario.

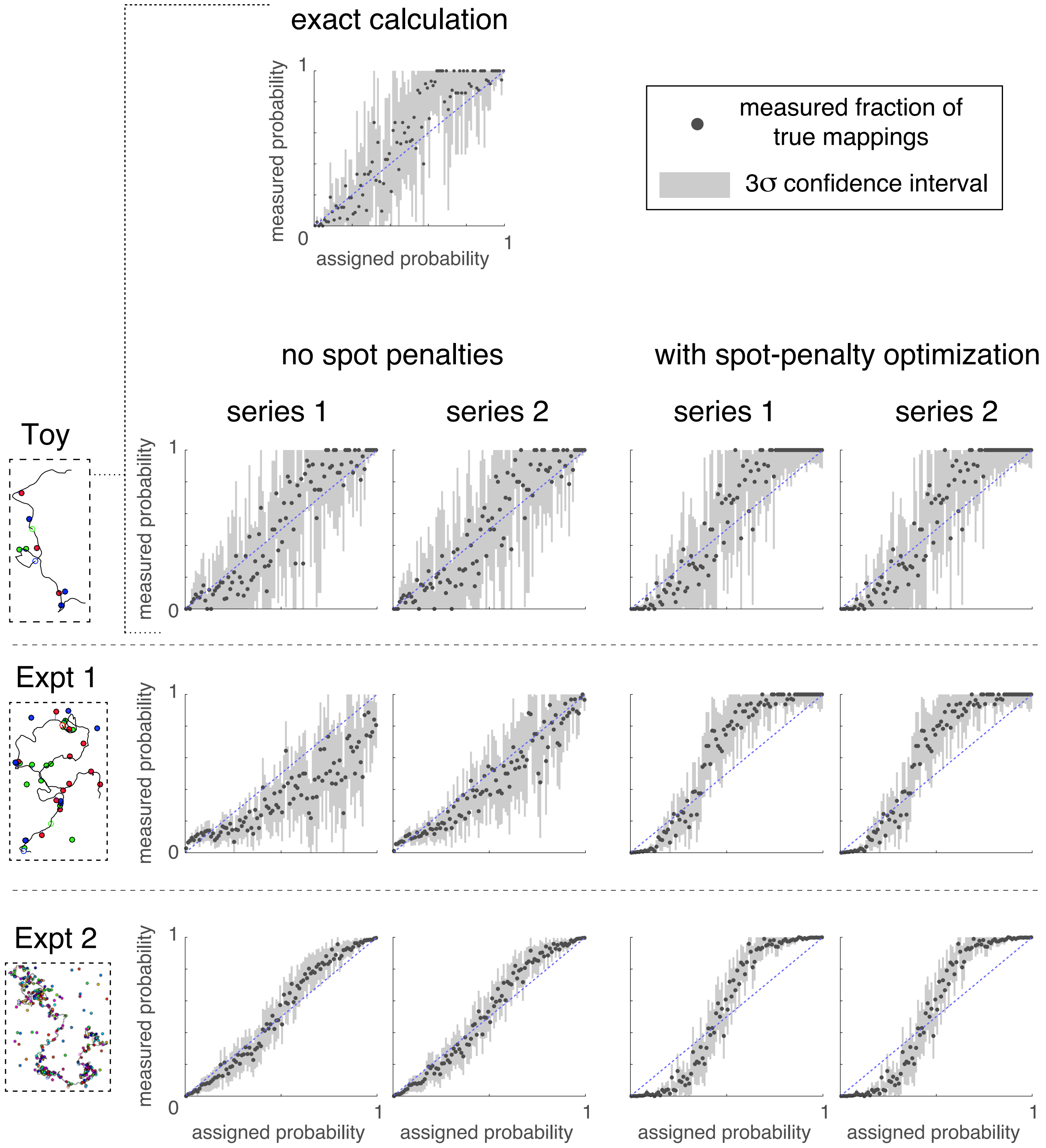

Figure S3. Accuracy of mapping probabilities. Binned mapping probabilities (x axis) versus the fraction of true mappings in each bin (y axis), averaged over the various simulated experiments in each experimental scenario. Grey shaded regions show the 3σ range of uncertainty due to counting error.

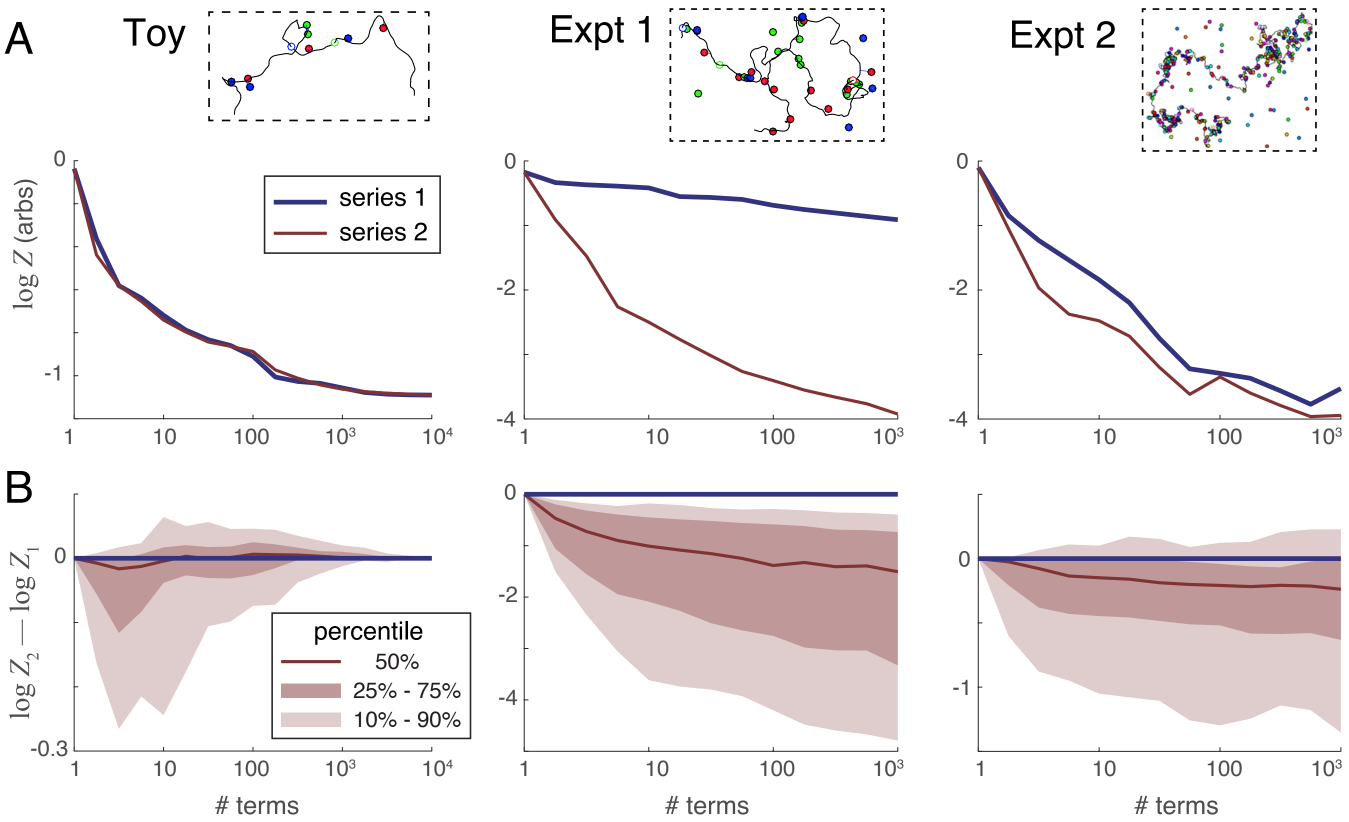

Figure S4. Partition function Z versus number of series terms. A. Relationship between $\log Z$ and the number of series terms for each simulated scenario, calculated as a median average of the relationships found in each of the 100 individual simulations in each scenario. **B.** Distributions of the difference between $\log Z$ calculated using series 2 and series 1. The fact that this quantity is generally negative when some but not all series terms are included reflects the fact that series 2 removes illegal conformations from Z faster than series 1.
