# Supplementary material for: Improved inference of chromosome conformation from images of labeled loci
Source: F1000Res. 2019 Mar 28;7:ISCB Comm J-1521. Originally published 2018 Sep 21. [Version 3] doi: 10.12688/f1000research.16252.3 (PMC6644830; doi:10.12688/f1000research.16252.3)
Supplement: Peer review history

# Peer review: Reviewer response for version 3

**DOI:** 10.5256/f1000research.20533.r46353

**Published:** 2019-07-19

**Authors:** Marco Cosentino Lagomarsino, Vittore Scolari

**Affiliations:** 1 Physics Department, University of Milan, Milan, Italy; 2 IFOM Foundation—FIRC Institute of Molecular Oncology, Milan, Italy; 3 Pasteur Institute, Paris, France

We have now both read the replies and the new manuscript and we are satisfied.

We confirm that we have read this submission and believe that we have an appropriate level of expertise to confirm that it is of an acceptable scientific standard.

# Peer review: Reviewer response for version 2

**DOI:** 10.5256/f1000research.20223.r45547

**Published:** 2019-03-27

**Authors:** Ewan Birney, Carl Barton

**Affiliations:** 1 European Molecular Biology Laboratory, European Bioinformatics Institute (EMBL-EBI), Cambridge, UK

In my initial review of the paper "Improved inference of chromosome conformation from images of labeled loci" a few concerns were raised. My comments mainly related to putting the current work of the authors in better context with respect to the previous work as well as explaining a number of the details mentioned in the paper in more detail.

 I believe the authors have satisfactorily addressed my comments. They have added an appendix giving more information on the convergence properties of the used equations. They have addressed differences between align3d and previous work explaining how they are quite different approaches.

 Some concerns were also raised relating to the effect of the number of colors on reconstruction quality and they have added another paragraph explaining in more detail why this effect is seen. In addition to this it seems they have addressed or responded to all of the other reviewer comments.

 We are happy to approve indexing.

We confirm that we have read this submission and believe that we have an appropriate level of expertise to confirm that it is of an acceptable scientific standard.

# Peer review: Reviewer response for version 1

**DOI:** 10.5256/f1000research.17750.r40510

**Published:** 2018-12-10

**Authors:** Marco Cosentino Lagomarsino, Vittore Scolari

**Affiliations:** 1 Physics Department, University of Milan, Milan, Italy; 2 IFOM Foundation—FIRC Institute of Molecular Oncology, Milan, Italy; 3 Pasteur Institute, Paris, France

In the manuscript  “Improved inference of chromosome conformation from images of labeled loci", Ross and Costello present a computational inference method to reconstruct genome conformation from measurements of the positions of m labelled loci of known coordinates with n<<m colored fluorescent foci. The presented tool is a new version of their computational tool "align3d" with multiple improvements. The tool has the aim of inferring the polymer conformation of chromosomes in-vivo, starting from images of fluorescently-tagged genomic loci, where each color tags different loci at the same time.

 The authors provide a test of the algorithm with data that are generated computationally, in a simple (short polymers) or more complex (longer polymers, more colors) setting. Finally they provide a simplified (and limited - see point 5 below) test using data that are derived from empirical data.

 The question appears interesting due to its experimental motivation, although probably the method is not yet close to something with concrete applicability to experimental data. We think that this could develop into a useful tool for the global effort of understanding the chromosome conformation of organisms in-vivo. As physicists, we are concerned with some aspects related to the representation (modelling) of the polymer and the experimental situation. Our observations might be useful for the authors or for other scientists that intend to analyse this kind of experimental data.

  

1. The authors state that the inaccuracy of the DNA (conformation) model, i.e. how the physical distance of two loci scales with arclength distance along the genomic coordinate is a major factor of error (more precisely, this is a conditional distribution of distances given distance along the chain). They further state that nothing is known about this. However, this is not really the case, as both Hi-C and FiSH experiments with labelled loci give information about these quantities (Lagomarsino *et al.*, 2015 ^1^ and Fudenberg and Mirny, 2012 ^2^).
  In particular, the assumption that the polymer is a Gaussian chain seems very restrictive. A much less restrictive (though still limited) assumption would be that this scaling relation is a tuneable power-law. This assumption is particularly interesting because in this case the scaling law relating physical distance to distance along the genome is related to the contact probability measured in Hi-C data (Fudenberg and Mirny, 2012 ^2^). Indeed, in this scenario the contact probability (sometimes called “P(s)”, where s is the arclength distance) and the connection between genomic distance and typical spatial distance R(s) are related by a scaling (Polovnikov *et al.*, 2018 ^3^). Thus Hi-C data could be used to directly constrain the inference, or to compare with the results.
  In this last scenario one could use the inference to learn the scaling from data. It seems quite reasonable to us that this scaling should be one of the main observables to infer from the data. Imposing this scaling appears like imposing a specific behaviour on the configurations that we are attempting to infer. In this regard, one big question is whether the observable “scaling of physical distance with arclength distance” can be inferred from the data without making the problem under-determined. We would like to stimulate the authors to spend some words to address this question.
  As we suggest above, there are multiple possible approaches to this practical issue, such as the use of the observable quantity “P(s)”, the contact probability measured with Hi-C, or the use of an ansatz, such as a power law (Marie-Nelly *et al.*, 2014 ^4^), accompanied by a procedure to optimize the parameters.
2. The authors’ main hypothesis is that only one locus can map to each identified spot in the image, and, for this reason, the solution proposed is a heuristic method to solve the traveling salesman problem for the polymer on those loci. We observe that this might be a good practical assumption but it is not necessarily a good one for the chromosome, and for polymers in general. Polymers can have loops, even randomly. The definition of those loops depends on the resolution of observation (which experimentally will be limited by diffraction). The frequency of loops in chromosomes depends on important physical and biological parameters such as active looping (Fudenberg *et al.*, 2016 ^5^), the presence of different solvent phases and the balance between steric and other kinds of interactions (Scolari and Lagomarsino, 2015 ^6^) as well as from steps of the experimental protocols (Scolari *et al.*, 2018 ^7^). Hi-C experiments, measure loops and quantify their specific and generic properties. In terms of the genomic distance, it has been shown that at small distances the chromosomes are very compact, and the amount of this compaction varies widely across conditions (Lazar-Stefanita *et al.,* 2017 ^8^ and Muller *et al.,* 2018 ^9^) even for the same organism. For increasingly longer distances, generally, the probability of making a loop normally decreases monotonically with genomic distance. Thus, we think that the authors’ approach should be applicable to an increasing number of cases by increasing the scale of observation and modelling, under the condition that the relation that ties the genomic distance to the three-dimensional distance is chosen correctly.
3. The algorithm is focused on a single chain conformation and does not exploit ensembles. Typically in such experiments one expects to have fairly low precision of localisation, but almost arbitrarily large amount of realisations (different cells). Each will be different but will also have common properties, and relaxing the question could make the inference process much easier. After all, inferring precisely a single configuration is not so relevant, because it will change in time due to natural fluctuations of the system. It is more useful (and well defined) to infer some ensemble properties (at fixed conditions for the cells such as time and phase into the cell cycle), and then quantify the cell-to-cell diversity with respect to such average behavior.
4. These images will come from microscopy and they will likely be 2D projections, or have lower resolution in the z direction. The authors do not address this issue (and in general the issue of resolution seems underestimated), but we expect it to be quite important in any concrete situation.
5. In regard to the final example, we notice that the data is binned at 1mb and then interpolated at 100kb with a spline, we wonder if this resolution improvement introduces any alterations in the reconstructed conformations of the polymer. For this reason, it seems reasonable to perform a more thoughtful statistical analysis with different levels of interpolation to support this choice.

We confirm that we have read this submission and believe that we have an appropriate level of expertise to confirm that it is of an acceptable scientific standard, however we have significant reservations, as outlined above.

## References

[1] : From structure to function of bacterial chromosomes: Evolutionary perspectives and ideas for new experiments. *FEBS Lett*.2015;589(20 Pt A) : 10.1016/j.febslet.2015.07.002 2996-3004 10.1016/j.febslet.2015.07.002 26171924

[2] : Higher-order chromatin structure: bridging physics and biology. *Curr Opin Genet Dev*.2012;22(2) : 10.1016/j.gde.2012.01.006 115-24 10.1016/j.gde.2012.01.006 22360992PMC3697851

[3] : Fractal Folding and Medium Viscoelasticity Contribute Jointly to Chromosome Dynamics. *Phys Rev Lett*.2018;120(8) : 10.1103/PhysRevLett.120.088101 088101 10.1103/PhysRevLett.120.088101 29542996

[4] : High-quality genome (re)assembly using chromosomal contact data. *Nat Commun*.2014;5: 10.1038/ncomms6695 5695 10.1038/ncomms6695 25517223PMC4284522

[5] : Formation of Chromosomal Domains by Loop Extrusion. *Cell Reports*.2016;15(9) : 10.1016/j.celrep.2016.04.085 2038-2049 10.1016/j.celrep.2016.04.085 PMC488951327210764

[6] : Combined collapse by bridging and self-adhesion in a prototypical polymer model inspired by the bacterial nucleoid. *Soft Matter*.2015;11(9) : 10.1039/c4sm02434f 1677-87 10.1039/c4sm02434f 25532064

[7] : Kinetic Signature of Cooperativity in the Irreversible Collapse of a Polymer. *Phys Rev Lett*.2018;121(5) : 10.1103/PhysRevLett.121.057801 057801 10.1103/PhysRevLett.121.057801 30118310

[8] : Cohesins and condensins orchestrate the 4D dynamics of yeast chromosomes during the cell cycle. *The EMBO Journal*.2017;36(18) : 10.15252/embj.201797342 2684-2697 10.15252/embj.201797342 PMC559979528729434

[9] : Characterizing meiotic chromosomes' structure and pairing using a designer sequence optimized for Hi-C. *Mol Syst Biol*.2018;14(7) : 10.15252/msb.20188293 e8293 10.15252/msb.20188293 30012718PMC6047084

# Response

**Published:** 2019-03-11

**Authors:** Brian Ross

**Affiliations:** University of Colorado Anschutz Medical Campus, USA

We wish to thank Reviewers 2 for their many helpful comments and insights. To address their comments as well as several concerns of our own, we have made a number of changes to our analysis, our results and the content of the main paper, and added a new appendix. The changes made to the code required us to regenerate all 9 figures that show results, although only Figure 7 changed significantly. We have also updated our GitHub repository containing all our code and example data.

Reviewers 2 pointed out that the we were too strong in our language stating that 'nothing is known' about the DNA model. We agree and we have removed this wording from the last paragraph in the Introduction. They also suggest the use of contact probabilities as a basis for inferring the distance function used in the model. This would be possible, but there is actually a direct measurement of the distance function (Wang *et al.,* 2016) which we have decided to use instead. Wang *et al.* found a 1/3 scaling exponent between L and R at the relevant length range, which we incorporated into our model for analyzing the Hi-C reconstruction (but not the random chains, whose exponent is 1/2) and then reran the results shown in Figure 7. The results did not change very much because most adjacent loci are close enough that R = k*L, i.e. the exponent is 1. The reviewers point out this could be due to our spline interpolation used to increase the resolution of the conformation. Unfortunately we were not able to directly infer higher-resolution conformations because the Hi-C data set recommended by Zhu *et al.,* 2018 (the Hi-C inference method called 'GEM') is binned at 1 Mb resolution, and this sets the resolution of the GEM conformations. We do not believe this is a problem for 2 reasons: 1) our average label spacing is ~2/3 Mb, not far below the 1 Mb GEM conformation subunit length, and 2) the inferred conformations seem to have a persistence length somewhat above the length of a subunit, although we cannot rule out that this might change with a different bin density. We agree that it would be ideal to have a higher-resolution structure (although bin sampling would become an issue using this Hi-C data set), but we suspect that the errors in Hi-C inferences probably overwhelm the resolution issue.

We want to point out that our use of a Gaussian chain for reconstruction (but not for producing the DNA chains) is not incompatible with the scaling relations mentioned by Reviewer 2, because these scaling relations determine average spatial separation R of two loci based on their genomic separation L, but not the form of the distribution p(r|R). We have chosen to model p(r|R) with a Gaussian (partly for convenience since it is easy to factor in localization error, but partly for other reasons; see below) having = R^2, but R is in turn calculated as R = L^power. In our earlier draft this power was fixed at 1/2 for distances above a persistence length, but as Reviewers 2 pointed out recent experimental data show exponents of 1/3 - 1/5. To address this issue, we generalized our program to accept more general DNA models consisting of different power laws at different inter-locus distance regimes, and our new results use exponents of either 1/2 and 1/3 for long DNA segments, depending on the simulated experiment. To make this clearer, we have added a new paragraph to the Results section (2nd paragraph) explaining the model selection in our simulations, as well as how a model would be chosen in a real experiment.

In our initial submission we claimed that a systematic error seen in the mapping probabilities was due to overestimation of the missing-spot rate. Since then we have both fixed the missing-spot rate estimation and made major progress in figuring out the real cause of the error, which we explain in detail in a new Appendix 3 and refer to in several places in the text.  The error comes from the fact that the Gaussian chain model used for reconstruction differs significantly from the wormlike chain model used to generate our simulated contours. While Reviewers 2 were concerned that the use of a 'wrong' model would skew the results, we believe that the opposite interpretation is more accurate: the fact that we obtain high-quality results even when the reconstruction model differs from the model used to produce the conformations shows that our approach is robust to model error. Appendix 3 justifies this intuition, by showing that model error causes our results to appear less certain than they are, but does not cause reconstruction errors if the reconstruction model is less sharply-peaked than the true model. This is the other justification for using the Gaussian chain model, which is quite permissive of unexpected behavior that we may find given that the true *in-vivo* DNA model may behave unexpectedly sometimes which may be very difficult to measure exactly in calibration experiments. We have also added a new 3rd paragraph to the Discussion explaining this.

We agree with Reviewers 2 that loops certainly can happen and, to the extent that they can be distinguished by microscopy, our algorithm is certainly capable of finding looped conformations (even if the loop is over 2 adjacent loci -- our Gaussian model peaks at r = 0).  If two loci of the same color happen to overlap in a microscope image, one may be missed -- this is considered a missing-spot or false-negative error, as mentioned in the footnote in the Introduction. Since our algorithm is capable of handling both false negatives and false positives (extra unbound spots), we do not anticipate loops to be a problem. If there are many points of overlap coming from an identical color sequence (e.g. if two copies of the same chromosome overlap) then the reconstruction fragments can become fragmented, with ambiguity as to which piece goes with which other piece -- we have added a brief note about this to the Discussion section.

Reviewers 2 point out that align3d is a single-cell method, not an ensemble method, and we completely agree. We believe that aggregating single-cell conformations will give many interesting insights that one could not get by aggregating, for example, pairwise distances.  Our method should be seen as one possible means of obtaining these cell conformations.

Finally, Reviewers 2 raise the issue of resolution in the z dimension: we certainly do consider localization error in z, both in generating the spot localizations (which have z error as well as x/y error) and in the reconstructions (where the errors in x/y/z are required inputs). In all simulations we set the z localization error higher than x/y error (200 vs 100 nm in normal resolution, 50 vs 30 nm in superresolution), reflecting the fact that axial resolution is worse in most setups. We have updated the main text to more explicitly give the localization error in the various simulations.

References:

Wang, S., Su, J. H., Beliveau, B. J., Bintu, B., Moffitt, J. R., Wu, C. T., & Zhuang, X. (2016). Spatial organization of chromatin domains and compartments in single chromosomes. *Science*, *353*(6299), 598-602.

Zhu, G., Deng, W., Hu, H., Ma, R., Zhang, S., Yang, J., ... & Zeng, J. (2018). Reconstructing spatial organizations of chromosomes through manifold learning. *Nucleic acids research*, *46*(8), e50-e50.

# Peer review: Reviewer response for version 1

**DOI:** 10.5256/f1000research.17750.r38643

**Published:** 2018-11-07

**Authors:** Ewan Birney, Carl Barton

**Affiliations:** 1 European Molecular Biology Laboratory, European Bioinformatics Institute (EMBL-EBI), Cambridge, UK

In this paper the authors update and build on a method they have previously published known as ‘align3d’. This method attempts to infer the chromosome conformation based on images of fluorescently tagged genomic loci. The authors claim that this updated method increases the accuracy of the inferred conformation as well as allowing the method to run on larger instances of the problem. They then go on to demonstrate where the method allows for the near perfect reconstruction of larger scale, simulated, labelled images. We believe that the article is worthy of indexing on the condition that some minor issues, outlined below, are addressed.

 In the introduction the authors mention a couple of other methods attempting to resolve similar problems. I think that this section should be expanded as there is no critical comparison of how this method compares to each of those mentioned. In particular the computational methods should be compared and contrasted so it is clear to the reader how this method differs from others.

 Whilst reviewing this paper we were unable to access the supplementary data. This must be made available before the paper can be indexed. Some things the authors could include in the supplementary section that would be useful from the computational perspective would be the type of series expansion being used and any information on how quickly the series expansion converge to the original formula. The authors have experimentally checked on the convergence properties but sometimes it's quite simple to determine theoretically how quickly some approximation converges. This information could be useful in determining better expansions and would explain more concretely why they get some of the results they see.

 In the experimental section of the paper the authors generate three different types of simulation that they denote 'Toy', 'Experiment 1' and 'Experiment 2'. In the discussion of the results the authors make the following comment:

 ‘Use of more colors dramatically improves reconstructions. Our most striking result is that simulations of the ambitious Experiment 2 produce far better results than even the Toy scenario, despite the fact that these simulations have more loci per color than either the Toy scenario or Experiment 1. This can be seen in the amount of unrecovered information shown in the simulation-averaged plots of Figure 5A. Thus a push to 20-color labeling could prove critical for genomic reconstruction at the chromosome scale and beyond. At the end of this section we revisit Experiment 2, in order to assess the reconstruction quality when analyzing more realistic DNA contours having tighter confinement.’

 The authors should make some attempt to explain this situation. Actually if you increase the number of colours and also increase the number of loci with the same colour then you would not obviously assume that the problem should be harder. It very much depends on how each is increased within proportion to each other.

 An increase in the number of unique colours available should lead to the problem being exponentially easier as you are effectively exponentially decreasing the ambiguity in the data set. Should you also increase the number of loci labelled with the same colour then you wouldn't expect the problem to become harder unless that increase was large enough to outweigh the effects of the increase in the number of available colours. In this sense it could be argued that many instances of the 'Toy' example are fundamentally more challenging than the (on the face of it) more complicated 'Experiment 2'. This should somehow be addressed by the authors.

 Also in the discussion of the experimental results the authors note that '20-color labelling leads to near-perfect reconstructions.' This result is consistent with our results reported by Barton *et al. *(2017 ^1^). It would be good to mention this as although the computational methods are different, the simulations are generated in different ways and the resolution simulated is different, both methods suggest that if ~20 colours are available then near perfect reconstruction is possible. The authors should also point out the similarity of the number of colours needed in both their method and ours.

 The differences in computational methodology yet similarity in the numbers of colours needed for near perfect reconstruction perhaps suggests to me that both methods are in some sense 'naive'. There must exist a minimum number of colours required for a certain average reconstruction performance (with the appropriate caveats) but we would be surprised if it was as high as 20. It could be interesting to see the authors add some discussion about this connection and any insight they might have into it.

 Finally there have been a number of different attempts to simulate super resolved images of the type used in this and other computational methods. If the authors can use this data as input or the data can easily be coerced into an appropriate format for this method then the paper would be much stronger with the addition of results of using the method against these datasets. In this way the authors can clearly demonstrate that the method they propose is not simply good on their own simulated data, but also performs robustly on other independently generated simulations.

We confirm that we have read this submission and believe that we have an appropriate level of expertise to confirm that it is of an acceptable scientific standard, however we have significant reservations, as outlined above.

## References

[1] : ChromoTrace: Computational reconstruction of 3D chromosome configurations for super-resolution microscopy. *PLoS Comput Biol*.14(3) : 10.1371/journal.pcbi.1006002 e1006002 10.1371/journal.pcbi.1006002 29522506PMC5862484

# Response

**Published:** 2019-03-11

**Authors:** Brian Ross

**Affiliations:** University of Colorado Anschutz Medical Campus, USA

We wish to thank Reviewers 1 for their many helpful comments and insights. Based on these comments and those of Reviewers 2, we have made some changes to our analysis, updated our results (particularly those shown in Figure 7) and the content of the main paper, and added a new appendix.

We apologize for the problems Reviewers 1 had in accessing the Supplemental Material. The material was uploaded and available (to us), but the 'Appendix x' links lead nowhere in the published version. These seemingly dead links have been removed.

Reviewers 1 suggested that in the Introduction we compare our align3d method to the other published method that we are aware of (ChromoTrace) to highlight their differences. We agree that this is indeed a useful addition, and so we have added several sentences to the Introduction (2nd paragraph) contrasting the two algorithms. We are not experts on ChromoTrace, and if we have mischaracterized it in some way we apologize and hope the reviewers will correct us.

Reviewers 1 also inquired about the exact series expansion formulas we used. The expansion formulas are in the Methods section of the main text, not the Supplemental material. To make this clearer we have added an equation number to the series definition preceding the coefficient formulas (this is the new Equation 3), and referenced that equation explicitly in the two coefficient formulas (which are now Equations 4-5). Thus the series definitions are fully in the main body of the paper, and only their derivations are in Appendix 2.

One technical detail is that our original code could not use our series expansions in conjunction with the preexisting capability to 'fix' certain loci to map to certain spots in the image, in order to obtain mapping probabilities that are conditional on the fixed loci. This has been addressed in the new version of the code. This oversight did not affect the results shown in the paper, but it did require us to add a explanatory paragraph to the end of Appendix 2.

Reviewers 1 asked about the finding that our simulated Experiment 2 reconstructions came out much better than the Experiment 1 reconstructions, despite having more labeled loci of a given color. We have added several sentences to the Results section ("Use of more colors dramatically improves reconstructions" section) explaining that we believe that it is the spatial density of labeled loci rather than the absolute number that determines the reconstruction quality. Reviewers 1 noticed the same finding in Barton *et al.* (2018); we have added this citation. We have not systematically tested performance as a function of the number of colors; we chose 20 simply based on the fact that 10 sequential hybridizations is reasonable for our planned experiment based on conversations with our collaborator (Wang *et al.,* 2016, demonstrate 17 rounds). Since we haven't noticed a plateau in reconstruction performance versus number of colors, as evidenced by the fact that the 20-color reconstructions still have some uncertainty, we do not see a reason to go towards fewer colors.

A final question raised by Reviewers 1 concerned the issue of superresolution in the simulated images. Since our spots are presumed well-separated (based on the data of Wang *et al., *2016) we believe we can get super-resolved spot localization without having to use special microscopes or fluorophores, and without having to resolve individual fluorophores. Thus the superresolution comes for free on normal images at the scale we consider here. We have added text explaining this (new final paragraph of Results), and also a second set of conformational reconstructions to Figure 7 showing explicitly the benefit of superresolving the spot locations. If we were to push to higher-genomic-resolution labeling (say, 10s-100s kb locus separation; current simulations are at ~600 kb) then we would indeed need superresolution microscopes, but since those are not the experiments simulated here we did not try to simulate those images. In fact this is why we chose to label these simulations at the 600 kb resolution.

Although we were not able to increase the Hi-C inferred resolution, we did discover that we had misinterpreted the scale of the Hi-C-derived conformations of Figure 7, thus underestimating the relative magnitude of microscope error in these simulations. Our new plots have corrected this error. Owing to the larger microscope error our new reconstruction quality is somewhat worse as measured by our information metric. To compensate we improved our script that estimates a likely conformation from our output (mapping p-values), and as a result these likely conformations are roughly of the same quality as before. We also added a parallel set of superresolution reconstructions to this figure, in order to show explicitly the benefit of reducing microscope error.

References:

Barton, C., Morganella, S., Oedegaard, O., Alexander, S., Ries, J., Fitzgerald, T., ... & Birney, E. (2018). Chromotrace: reconstruction of 3D chromosome configurations by super-resolution microscopy. *bioRxiv*, 115436.

Wang, S., Su, J. H., Beliveau, B. J., Bintu, B., Moffitt, J. R., Wu, C. T., & Zhuang, X. (2016). Spatial organization of chromatin domains and compartments in single chromosomes. *Science*, *353*(6299), 598-602.

Zhu, G., Deng, W., Hu, H., Ma, R., Zhang, S., Yang, J., ... & Zeng, J. (2018). Reconstructing spatial organizations of chromosomes through manifold learning. *Nucleic acids research*, *46*(8), e50-e50.
